# Comparative epidemiology and preparedness for African swine fever and porcine reproductive and respiratory syndrome in four major Asian swine industries

**DOI:** 10.3389/fvets.2026.1888726

**Published:** 2026-07-20

**Authors:** Thanh Che Nguyen, Nakarin Pamornchainavakul, Kimberly VanderWaal, Roongtham Kedkovid, Roongroje Thanawongnuwech

**Affiliations:** 1 Department of Veterinary Pathology, Faculty of Veterinary Science, Chulalongkorn University, Bangkok, Thailand; 2 Department of Veterinary Population Medicine, College of Veterinary Medicine, University of Minnesota, St. Paul, MN, United States; 3 Department of Veterinary Medicine, Faculty of Veterinary Science, Chulalongkorn University, Bangkok, Thailand

**Keywords:** African swine fever, Asia-Pacific, biosecurity, epidemiology, porcine reproductive and respiratory syndrome, preparedness, surveillance, transboundary animal diseases

## Abstract

East and Southeast Asia account for over half of global pork production but face unprecedented challenges from transboundary animal diseases (TADs). African swine fever (ASF) and porcine reproductive and respiratory syndrome (PRRS) are two of the most consequential diseases reshaping the region's swine industry. This narrative review summarizes literature and institutional reports from peer-reviewed scientific databases to provide a clear comparative epidemiological overview of these diseases across a strategic cross-section of four major producers: China, Vietnam, Thailand, and the Philippines. While both viruses exploit identical regional vulnerabilities, such as high-density smallholder networks, porous border trade, and farm-level containment gaps, their transmission characteristics and evolutionary patterns differ fundamentally. Driven by extreme environmental resistance, the ASF virus operates primarily via human-mediated, anthropogenic routes and has evolved from causing acute epidemics to an unpredictable endemic state marked by recombinant and gene-deleted strains. Conversely, the highly dynamic, rapidly evolving PRRS virus acts as a self-sustaining endemic engine driven by efficient host persistence, long-distance aerosol spread, and immune-evasive genetic diversification. Furthermore, national control strategies vary widely based on production infrastructure, ranging from China's mega-industrialization and precision culling to Vietnam's reliance on partial live-attenuated vaccination, Thailand's strict physical biosecurity, and the Philippines's archipelagic zoning. Together, these approaches highlight that preparedness and disease control in each country depend on harmonized strategies, upgraded biosecurity standards, knowledge sharing among the stakeholders, and transparent surveillance aligned with the World Organization for Animal Health guidelines. Importantly, cross-learning between ASF and PRRS, as well as among other TADs' frameworks, offers a pathway to more resilient not only national but also regional food security. Ultimately, advancing transboundary preparedness in the region requires coordinated, multi-country collaboration that integrates molecular surveillance networks and validated immunization protocols with practical, system-adapted execution.

## Graphical Abstract

**Figure:**
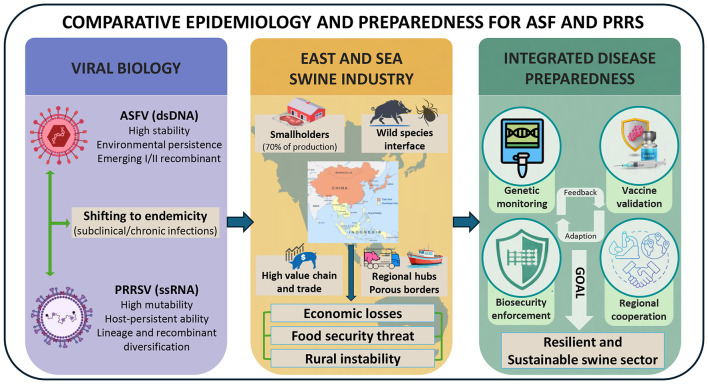

## 1 Introduction

The global swine industry faces increasing threats from viral transboundary animal diseases (TADs) that compromise food security, economic stability, and environmental sustainability (1). East Asia and Southeast Asia (SEA) have emerged as a major epicenter for these epizootics due to highly concentrated swine sectors and extensive, porous trade networks (2). This combined region houses over 50% of the global porcine population across a wide range of production systems, from traditional smallholders and backyard pens to medium- to large-scale, semi-commercial, and modernly integrated industrial operations (3). These systems remain highly susceptible to economic disruptions and market instability caused by co-circulating pathogens, resource constraints, and variable management policies. This vulnerability has become particularly acute due to the epidemiology overlap between the devastating panzootic invasion of African swine fever (ASF) and the long-standing endemicity of porcine reproductive and respiratory syndrome (PRRS) (4–6).

These two diseases present distinct biological and epidemiological challenges. ASF is a reportable disease to the World Organization for Animal Health (WOAH), characterized by highly infectious but not contagious, sudden death with fatality rates approaching 100% in acute cases (7). Its causative agent, African swine fever virus (ASFV; family *Asfarviridae*, genus *Asfivirus*), is a giant, double-stranded DNA (dsDNA) virus that exhibits extreme environmental persistence (8). In the Asia-Pacific region, ASF was first detected in China in 2018 and subsequently spread to neighboring countries, reaching 20 countries by 2026 (9). Conversely, PRRS is caused by the highly mutable positive-sense single-stranded RNA (ssRNA) virus, porcine reproductive and respiratory syndrome virus (PRRSV; family *Arteriviridae*, genus *Betaarterivirus*) (10). The first serological detection of PRRSV in Asia occurred in the East Asian region as early as 1985 (11). Since then, PRRS has become an endemic burden marked by severe reproductive failure in breeding stock and respiratory distress in growing pigs (10). Despite fundamental genomic differences and contrasting clinical timelines, both viruses share a common macro-epidemiological pathway: their circulation across East and SEA swine industries is driven by structural vulnerabilities in the regional live-animal transport network and localized deficiencies in farm-level biosecurity (4, 6).

While existing literature frequently addresses these pathogens in isolation, a critical research gap remains regarding how a stable DNA virus (ASFV) and a highly mutable RNA virus (PRRSV) concurrently exploit identical systemic vulnerabilities within transitioning agricultural economies. Comparing these two pathogens could provide a valuable epidemiological baseline that allows researchers to dissect how differing viral survival strategies capitalize on the same value-chain weakness, thereby revealing whether biosecurity interventions designed for acute epizootics are effective against chronic, endemic strains. To investigate this co-circulation dynamic, this review focuses on a strategic cross-section of leading East and SEA pork producers: China, Thailand, Vietnam, and the Philippines. These four nations provide an ideal comparative foundation because they represent a diverse spectrum of production archetypes, ranging from China's rapid mega-industrialization to the fragmented, smallholder-dominated networks of Thailand, Vietnam, and the Philippines. All have shared experiences of severe, overlapping ASF and PRRS outbreaks that have forced shifts in national biosecurity policies.

This narrative review synthesizes current epidemiological data and biosecurity frameworks across these four pivotal pork producers. To clarify the manuscript's trajectory, our specific objectives are to: (i) characterize and contrast the molecular and transmission mechanisms of ASFV and PRRSV within high-density networks; (ii) discuss how regional variations in pig housing, physical geography, and live-animal marketing structures (supply chain, transaction hubs, and trading routes) accelerate transboundary viral dissemination have recently changed; and (iii) summarize the successes and failures of national control strategies, such as compartment systems, zoning, and vaccination guidelines. Ultimately, this review aims to provide a comparative perspective on transboundary swine disease preparedness that accounts for diverse socio-economic and structural contexts across transitioning swine industries.

## 2 Literature search and selection strategy

To ensure a transparent and reproducible synthesis, a systematic literature search was conducted across the PubMed, Scopus, and Google Scholar databases, supplemented by official situation reports from WOAH, Food and Agriculture Organization (FAO), and national authorities. The search architecture with keywords: (“African swine fever” OR “ASF” OR “porcine reproductive and respiratory syndrome” OR “PRRS”) AND (“China” OR “Thailand” OR “Vietnam” OR “the Philippines”) AND (“epidemiology” OR “transmission” OR “biosecurity” OR “control strategies” OR “persistence”). Studies were included if they provided primary epidemiological data, molecular lineage characterization, or national policy appraisal for the target countries. Exclusion criteria removed non-English publications, duplicates, and papers lacking specific regional or mechanistic data.

## 3 East and Southeast Asian swine production systems, culinary culture, and geographic topographies pose risk factors for TADs

### 3.1 Pork production capacity

**Figure 1:**
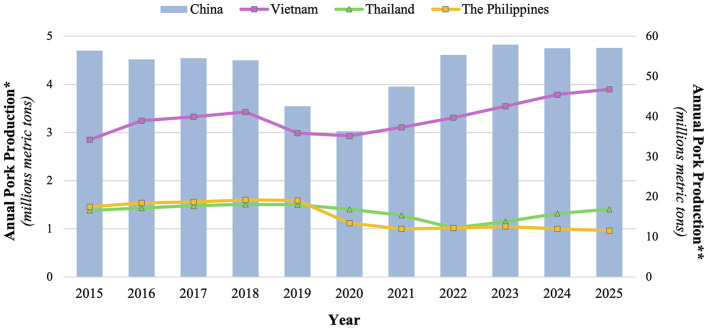
The summary of total annual pork production in China, Vietnam, Thailand, and the Philippines from 2015 to 2025. Data source is from Production, Supply, and Distribution (PS&D) on the United States Department of Agriculture (USDA).

On a global scale, East Asia and SEA dominate pork production and consumption. Driven by massive industrial capacity and rapid intensification, China alone accounts for approximately 49% of total global pork production by volume, yielding over 57 million tons annually. Within the SEA sub-region, production metrics reflect highly varied national capacities and distinct demographic scales. Vietnam leads sub-regional production at 3.9 million metric tons, followed by Thailand at 1.41 million metric tons, where the national herd is projected to reach 23.58 million heads in 2026 to support its role as a major regional exporter of chilled and processed pork (12). Conversely, due to ongoing animal health challenges, Filipino pork production contracted slightly, reaching 960,000 metric tons in 2025 (Figure 1). In most countries, such as China and Vietnam, output is heavily sustained by dense domestic markets with high per capita consumption rates, recorded at 29.5 and 24.5 kg in 2025, respectively (13). In short, the sheer volume of regional pork production creates high-density environments, while intensive consumer demand drives extensive animal transport networks and swill-feeding cycles that act as primary risk factors in the dissemination of TADs.

### 3.2 Characterization of pig housing

Swine production systems across the four analyzed countries share epidemiological landscapes defined by limited zoning, extensive animal movement, and heterogeneous biosecurity practices. Production is historically characterized by a dominance of smallholders and backyard farms situated in close peri-urban proximity to large-scale commercial operations (3). However, the formal definitions and contributions of small-scale farms vary significantly across national jurisdictions, hindering direct comparisons unless standardized by market share and capacity thresholds. Specifically, small-scale farms with < 20 pigs in the Philippines and Vietnam, < 50 pigs in Thailand, and < 500 pigs slaughtered annually in China constitute a major proportion (Table 1) (14–17). Despite this, this system shares a varied portion in the total annual pork production: China (~ 40%), Vietnam (35%−40%), Thailand (10%−15%), and the Philippines (over 60%) (3, 18–20). Smallholders often lack the financial and technological capacity to implement rigorous biosecurity and modern livestock management practices, thereby exacerbating risks. Biosecurity, in particular, includes internal controls to limit pathogen spread within a farm and external controls to prevent the introduction of new pathogens into the farm (21). Thus, they frequently serve as initial outbreak sites and long-term viral reservoirs, particularly when herd depopulation and fallow periods are economically unfeasible for livelihood-dependent smallholders, leading to recurrent outbreaks (22).

**Table 1:** Standardized national definitions and market contributions of small-scale swine farms in the four analyzed countries.

Country	Small-scale definition	National inventory (%)	Market contribution (%)	Reference
China	<500 pigs slaughtered annually	~99^*^	~40	(3, 16)
Vietnam	<20 pigs per inventory cycle	64−70^*^	35−40	(14, 19)
Thailand	<50 pigs per inventory cycle	~91^*^	10−15	(15, 20)
The Philippines	<20 pigs per inventory cycle	~67−83^**^	~60	(17, 18)

^(*)^: of total registered pig farms; ^(**)^: of total swine population.

The intermingling of traditional farms and industrialized units creates a dual-risk system: household farms facilitate viral maintenance while large farms enable rapid amplification and regional dissemination. As noted by Lamberga et al. (23), the long high-risk period (HRP, the time between virus introduction and detection) in large commercial units indicates extensive silent disease spread. Thus, a single breach in a high-capacity facility results in far more catastrophic regional dissemination than a localized breach in a small-scale unit, leading to the loss of a large number of animals. To be more supportive, the basic *R*_0_ reproduction number estimates the average number of secondary cases directly generated by one case in a population, assuming that all individuals (within or between herds/farms) are susceptible. The *R*_0_ threshold is set at 1; when *R*_0_ < 1, the infection will eventually clear, whereas *R*_0_> 1 indicates the infection is potentially widespread (24). A systematic review summarized that the *R*_0_ within-herd transmission rate for Aujeszky's disease is 10, influenza A is 8.65, circovirus is 5.9, PRRS is 2.78, and ASF is 3.94, indicating these viral diseases are expected to spread rapidly within a herd (25).

**Figure 2:**
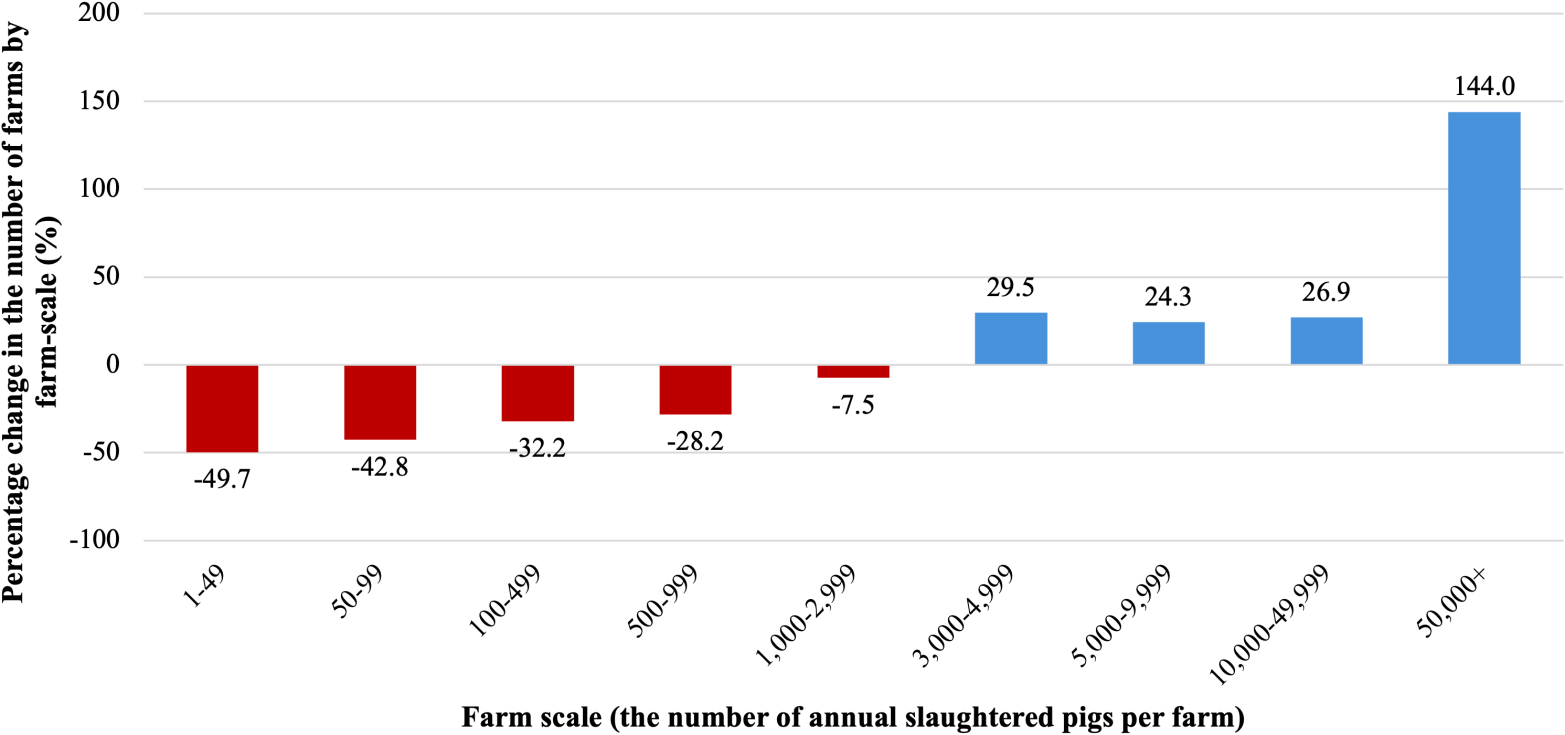
Trends in livestock farm model transformation in China between 2017 and 2022. Data adapted from the Ministry of Agriculture and Rural Affairs China. China animal husbandry and veterinary yearbook (2023).

Following recent ASF panzootic waves, China, Vietnam, and Thailand have gradually restructured their swine sectors. Biosecurity and environmental mandates have sidelined thousands of households and local slaughterhouses, favoring high-biosecurity industrial-scale production and modern abattoirs. Because countries have different data record systems, this review estimated different parameters to present this trend. China demonstrates the most obvious conversion, as measured by the total number of pigs slaughtered per farm (Figure 2). Vietnam has reduced the number of small-scale farms by 5%−7% annually, while increasing the volume of pork produced by commercial operations to 60%−65% (19). Thailand dropped almost 90,000 small-scale pig farmers compared to the pre-ASF outbreak period (26). Unlike these countries, the Philippines' situation has not undergone a structural transformation. While this shift has improved both awareness and animal husbandry practices, it has also introduced new risks. High-density populations, co-housing practices, and regional clustering of farms in high-production regions like Northeast Thailand and low-lying areas of Vietnam can amplify transmission once pathogens are introduced (27). Moreover, the expansion of industrial systems has outpaced veterinary infrastructure and surveillance capacity, leaving residual vulnerabilities (28).

### 3.3 Culinary and livestock culture

The farm-to-table value chain in East Asia and SEA is highly dynamic and often non-linear, known as fragmented, multi-directional trading networks with extensive intermediary dealers, shaped by strong cultural preferences for fresh pork (29). Daily slaughter and distribution through wet markets necessitate the continuous movement of live animals, increasing the risk of pathogen spread. During peak seasonal demand periods like the Tet/Lunar New Year, intensified transport and insufficient disinfection practices can transform vehicles and market environments into effective transmission hubs, recycling pathogens back into rural hinterlands and farm gates found in many Asian countries (30–32). Humans, such as farm workers, traders, and service personnel, act as mechanical vectors, transferring environmentally resistant pathogens via contaminated clothing, equipment, and vehicles. These human-mediated pathways can undermine even well-designed physical biosecurity barriers, especially in regions with high farm density and frequent inter-farm contact (30, 33). Continuous training, regular biosecurity drills, and targeted education programs for farm personnel are essential to foster behavioral change, enhance awareness, and promote a biosecurity-oriented culture, thereby improving the effectiveness and sustainability of biosecurity implementation.

Anthropogenic activities also foster pathogen-friendly ecosystems, rendering global eradication exceedingly challenging. Human movements, whether by international tourists or through humanitarian aid via ships or aircraft (34), coupled with expatriate behavior of transporting and consuming traditional food products (35), may pose a latent risk for long-distance disease transmissions. Furthermore, family-owned farmers often use irrigation systems or groundwater to clean enclosures or cool animals. Improper disposal of infected carcasses into rivers or shallow burial sites can contaminate natural water sources and soil with carcass-derived leachate, containing microorganisms, nutrients, and other pollutants. Such contamination may facilitate pathogen persistence in the environment, increase opportunities for indirect transmission to susceptible animals, and pose risks to public and environmental health by degrading water quality (36). For instance, a study in the United Kingdom reported that 212 contaminating surface and groundwater incidents were linked to six disposal sites for foot-and-mouth disease (FMD)-infected animals (37).

Commercial feed and ingredients further contribute to viral disease transmission. Viral persistence in feed matrices during transcontinental transport is well documented not only for PRRSV and ASFV but also for other TADs, including FMD virus, porcine epidemic diarrhea virus, and porcine circovirus type 2 (38). In Asia, local practices include drying grains on public roads, exposing feed to environmental contamination. Importing feed materials from endemic regions can also introduce inapparent infection directly into farm environments via the feed bin, bypassing perimeter biosecurity (39). Swill feeding practices persist among households despite regulatory restrictions, driven by economic pressures (40). This practice provides a direct transmission route for stable viruses, such as ASFV, to enter the swine population via raw or undercooked pork scraps (41). Moreover, swill feeding may induce gastrointestinal dysbiosis or chronic inflammation, potentially compromising the host's mucosal immunity and heightening susceptibility to co-infections (42).

The movement of high-value genetic materials, which may carry infectious pathogens, poses intricate biosecurity challenges. Industrial swine operations frequently import elite breeders and/or porcine semen from international or regional sources for rapid restocking (43). When sourced from areas with undetected infections, insufficient testing, and inadequate quarantine protocols, these materials become primary entry points for novel viral strains into naïve populations (44).

### 3.4 Geographic, climate topographies, and wild species

Asian swine industries' vulnerability to swine TADs stems not only from biological factors but also from physical geography. Mainland regions (China, Thailand, and Vietnam) are characterized by extensive land and river borders, with hundreds of unofficial crossing points that facilitate unregulated animal movement, trade, and human transport (45). For instance, to capitalize on price fluctuations, farmers frequently move infected live animals and contaminated products toward the border areas, acting as high-risk zones where the first viral outbreaks are commonly detected (46). In contrast, the Philippine archipelago of over 7,000 islands offers surrounding seas as natural barriers that should impede the spread. However, maritime trade and waste-disposal practices from international or inter-island shipping vessels circumvent these obstacles, enabling diseases to jump from island to island rather than advancing in a linear wave (47).

Climate conditions not only influence domestic swine facility management and disease control, but also furnish ideal habitats for wild suids (48). Tropical and subtropical environments require adequate ventilation and cooling infrastructure to mitigate heat stress and improve well-being in commercial systems (17). But in highland regions with low winter temperatures, these conditions are ideal for pathogens to persist in the environment, especially heat-sensitive viruses. Unlike domestic pigs confined to controlled pens, free-ranging wild boars present substantial challenges for effective culling and monitoring (49). Consequently, buffer zones between forested areas and livestock farms function as enduring reservoirs (50, 51). Pests and arthropods, such as birds, rodents, houseflies, mosquitoes, and ticks, serve as both biological and mechanical vectors in TAD transmissions (52). These wild species' interfaces complicate containment efforts, as surrounding landscapes may reintroduce pathogens into previously cleared farms.

## 4 Comparison between ASF and PRRS epidemiology in East and Southeast Asia

### 4.1 Viral biological features and evolution

ASFV and PRRSV diverge fundamentally in their structural, environmental, and evolutionary characteristics (Table 2). ASFV, the sole member of the family *Asfarviridae*, is an enveloped, multilayered, and large dsDNA arbovirus (53) characterized by exceptional environmental resilience. It remains infectious for months to years in inanimate objects such as feed, water, soil, fomites, and pork products, which serve as indirect transmission factors (33, 54). Transmission dynamics are complicated, with three major cycles: sylvatic, domestic, and wild boar, and involve several hosts: members of the *Suidae* family and soft ticks (*Ornithodoros* genus) (8, 53). At least 24 genotypes are classified based on the partial *B646L* (p72) gene sequence (55). ASFV has an estimated mean evolutionary rate of ~ 3.49 x 10^−6^ and ~ 1.31 x 10^−5^ substitutions/site/year (s/s/y), based on 71 and 42 whole-genome sequences (WGS) of genotype I (G.I) and genotype II (G.II), respectively (56, 57). G.II, which is highly genetically similar to the Georgia 2007/1 isolate, has been predominantly responsible for outbreaks in Asian countries (58). By 2022, G.I, recombinant between G.I and G.II (rI/II), and G.II-gene-deleted [multigene family (MGF) genes and/or the virulent *EP402R* gene] strains had emerged in China, indicating the virus's capacity for epidemiologically significant variation (59–62). At the moment, G.I circulates exclusively in China, while G.II-related isolates predominate in most Asian countries, with the rI/II exhibiting rising prevalence, as observed in Vietnam in 2023–2024 (63).

**Table 2:** Comparison of disease features between ASF and PRRS.

Features	ASF	PRRS
Causative agent	Large, enveloped dsDNA virus (*Asfarviridae*) (8)	Enveloped ssRNA virus (*Arteriviridae*) (10)
Genetic variability	Relatively stable (10^−6^-10^−5^ s/s/y) (56, 57)	Highly variable (10^−3^-10^−2^ s/s/y) (66–69)
Genetic diversity	At least 24 genotypes (55) Mutations and recombinations (61, 62, 91)	PRRSV-1 (3 or 4 subtypes) (75–77); PRRSV-2 (11 lineages, 23 sub-lineages, and variants) (66, 71–74) Mutations and recombinations (68, 155, 158)
Host range	Domestic pigs, wild boars, and soft ticks (*Ornithodoros* genus) (8)	Domestic pigs, wild boars, no biological vectors (10, 64)
Major transmission routes	Direct contact (blood-borne), human-mediated via contaminated environment and fomites, swill feed, pork products, limited aerosol, vector-borne (30, 33, 41, 87)	Direct contact (snout-to-snout), aerosol (long-distance), fomites, semen, vertical transmission (transplacental) (64, 103, 107)
Contagiousness	Moderate-high (depends on strain) Slow within-herd spread (*R_0_*= 1–3)^*^ (25, 159)	High (efficient airborne transmission) Rapid within-herd spread (*R_0_*= 2–5)^*^ (25, 64)
Environmental stability	Very high (survives long in meat, carcasses, cold conditions) (33, 54)	Moderate (survives long in cold but easily inactivated by disinfectants; prolonged persistence in live pigs) (64, 65)
Disease course	Peractute/Acute form (high-virulent infection) Subclinical/Chronic form (low-virulent infection) (84, 160)	Often endemic with recurring outbreaks (10, 95)
Time to peak transmission	Rapid in naïve herd with high-virulent strain infections (within 1–2 weeks), longer in endemic state (84, 160)	Slower build up at population level but continuous transmission cycle (months to years) (10, 95)
Mortality rate	Often very high (up to 90%−100% in acute outbreaks) (84, 160)	Variable (low-moderate; high in piglets with co-infection) (10, 95)
Economic impact	Catastrophic (mass culling, trade bans) e.g., US$200 billion in China (2018–2019) (140, 161, 162)	Chronic high losses (reduced productivity) e.g., US$200/sow/year in China (2014–2017) (98–100)
Epidemiology pattern	Epidemic waves in 2018, shifting to epidemicity (45, 113)	Endemic in most countries (96, 149)
Typical control goal	Eradication (where feasible) (163)	Management/mitigation (often living with the virus) (142)
Control strategy	Stamping-out, swill feed and movement control, biosecurity, surveillance, compartmentalization (22, 41, 128)	Herd stabilization, vaccination, internal biosecurity, monitoring, acclimatization, depopulation (101, 106, 143)
Diagnosis	Molecular detection (Antigen) (116)	Molecular, serology detection (Antigen, antibody) (119)
Vaccines	No widely available safe vaccine (especially in breeders) (131, 164)	Modified-live vaccines available (partial protection) (156, 165)

^*^: Under experimental settings.

In contrast to ASFV, PPRSV represents a hyper-mutagenic evolutionary paradigm. An enveloped, positive ssRNA virus in the family *Arteriviridae*, genus *Betaarterivirus*, PRRSV possesses low environmental stability and is highly sensitive to pH, temperature, ultraviolet light, and lipid-disrupting disinfectants (64), but may exhibit high persistence in hosts (65). However, driven by a low-fidelity RNA-dependent RNA polymerase, it achieves a high mean evolutionary rate, ranging from 10^−3^ to 10^−2^ s/s/y in the open reading frame 5 (ORF5) gene, fueling extensive genetic and antigenic diversity (66–69). It has been classified into two species: PRRSV-1 (*Betaarterivirus europensis*, predominant in Europe) and PRRSV-2 (*Betaarterivirus americense*, predominant in North America) based on WGS data or variability of most individual genes (70). To better understand, ORF5-based classification further divided PRRSV-2 into at least 12 lineages and 23 sub-lineages, and subsequently into variants (66, 71–74); meanwhile, PRRSV-1 consists of three or four subtypes: subtype 1 (1-Global, 1-Russian), subtype 2, and subtype 3 (75–77).

While PRRSV-2 historically dominated East and SEA swine industries, the contemporary landscape is defined by the extensive co-circulation and diversification of both species. PRRSV-2 evolution is driven by lineage 8 (highly pathogenic PRRSV, HP-PRRSV) and lineage 1 variants (NADC30-like and NADC34-like), which undergo frequent intra-lineage recombination, exacerbating nursery mortality (78–80). Concurrently, PRRSV-1 has diversified in these regions into distinct clades (i.e., BJEU06-1-like, HKEU16-like, and NMEU09-1-like), showing increased field virulence (81, 82). Crucially, pervasive recombination between wild-type field strains and modified-live vaccines (MLVs; e.g., Amervac-like revertants) occurs regularly (83). This intense flux generates substantial heterologous variation, severely undermining commercial vaccine cross-protection and driving recurrent, vaccine-escape outbreaks throughout the region.

### 4.2 Disease patterns and economic impacts

Generally, ASF and PRRS have distinct disease features and impacts on the East and SEA swine industry, though they are increasingly converging (Table 2).

ASF disease consists of various infection forms: hyperacute and acute forms with sudden death and near-100% fatality, which frequently begin in naïve populations. Whereas subacute and chronic forms are associated with low-virulence isolates, characterized by reproductive losses, lower mortality rates, and prolonged viral shedding (84). Once infection occurs, ASF naturally triggers sudden, extensive herd losses, subsequently devastating domestic pork supply and global protein markets. In the first epidemic year, China and Vietnam had lost 40% (130 million hogs) and nearly 25.5% (6 million culled pigs) of their national inventory herds, respectively (85, 86). This supply shock triggered a surge in pork prices, remediation costs, and environmental sanitation issues regarding the disposal of culled animals.

In 2018, ASF began its incursion into Asia, specifically China, marking a definitive turning point in global swine disease epidemiology. Over time, Vietnam and the Philippines officially reported index cases in 2019, and Thailand in early 2022 (9). Across these countries, several common epidemiological patterns emerge (Table 3):

(1) ASF dissemination across all settings is fundamentally shaped by shared structural vulnerabilities, including fragmented production systems, tightly interconnected value chains, human behaviors, extensive animal movement, uneven biosecurity, and limited veterinary resources for traceability (detailed in Section 3). These, collectively, enable short-term spread following the initial introduction in small family-owned backyards (87, 88).
(2) Three out of four countries, China in 2022, Vietnam in 2023, and Thailand in 2024, have detected high-lethality recombinant I/II in high-risk areas, such as the border and low-biosecurity-adapted farms (61, 63, 89). These continuous emergences and spreads again highlight the importance of cross-border transmission control among mainland countries.
(3) All countries have shown evidence of an epidemiological shift from acute epidemic outbreaks, caused by highly pathogenic genotype II strains, toward more unpredictable endemic dynamics. This evolution stems from the dynamic interplay of viral adaptation, restructuring of swine production, response strategies, and unresolved biosecurity deficiencies. It manifests as localized viral adaption, diversification, and regional dissemination, characterized by subclinical persistence and periodic re-emergence (90–94).

**Table 3:** Epidemiological patterns and control strategies for ASF and PRRS in China, Vietnam, Thailand, and the Philippines.

Criteria	China	Vietnam	Thailand	The Philippines
Primary pork production structure	Rapidly industrializing system with high animal densities and increasing vertical integration (16, 130)	Mixed system dominated by smallholders and backyard farms with fragmented value chains (14, 19)	Intermediate system with high-biosecurity commercial systems coexisting with smallholders (15)	Highly fragmented system with strong reliance on backyard farming (14, 17)
ASF status	Transitioning from acute epidemic shocks to a complex endemic state with merging low-virulence variants (34)	Complex endemic state with co-circulating low-virulent, rI/II, and vaccine-like strains (63, 90, 122)	Controlled endemic state with sporadic localized outbreaks. Hybrid rI/II, vaccine-like strain emergence (89, 123, 126)	Complex endemic state with recurrent outbreaks across major islands (138)
PRRS status	Endemic with high genetic diversity and ongoing viral evolution despite advanced control in industrial systems (144, 166)	Persistent endemicity driven by fragmented systems, variable vaccine use, and limited biosecurity (145)	Endemic but relatively better controlled in commercial sectors; Continuous circulation persists in mixed systems (165, 167)	Endemic with uneven control, driven by fragmented systems and inconsistent biosecurity implementation (147)
Primary ASF control strategy	Resilience-based: regional zoning, industrial consolidation, movement control, and precision culling (124, 127, 129)	Hybrid: combination of biosecurity measures and widespread use of LAVs (128, 162)	Containment-oriented: biosecurity-driven control with surveillance and movement restrictions, without vaccination (135)	Constraint-driven: shifting from formal eradication toward managed endemicity using zoning, vaccination, and partial biosecurity (17, 138)
Surveillance challenges	Reduced reliability of clinical detection due to low-virulence strains and subclinical infections (34, 129)	Managing vaccine-related evolutionary pressures and surveillance gaps in informal trade networks (162, 164)	Disease detection in smallholder sectors, wild boar and maintaining compliance across production systems (134)	Significant blind spots in informal markets, inter-island movement, and decentralized surveillance systems (140)

Unlike the abrupt epidemic crisis of ASF, PRRS embodies convergent endemic patterns after the acute outbreak in naïve populations, characterized by continuous circulation, high genetic diversity, and long-term reductions in suboptimal performance rather than catastrophic collapse (10, 95). Introduced in the late 20^th^ century, PRRS has exploited weaknesses in pig production systems and firmly established itself in most major pig-producing countries (96), resulting in varying levels of persistent endemicity, marked by sustained reductions in production performance (Table 3). Infection in breeding animals and at farrowing sites caused reproductive disorders, and a reduced number of weaned pigs, while infected growing-fattening pigs were susceptible to secondary infections, impairing growth rates and/or increasing mortality (97). For instance, the Chinese swine industry estimated an annual loss of CN¥1,424 (about US$200) per sow during 2014–2017 (98).

This prolonged burden is frequently underestimated at the national level in these countries. Since PRRSV is maintained through internal transmission and recurrent reintroduction, with evolutionary dynamics shaped by immune pressure and inconsistent vaccination in unstable herds, it subsequently accumulates over time, generating substantial economic costs and eroding overall system efficiency. Together, PRRS delineates baseline productivity in endemic regions and influences farm-level decision-making, biosecurity investments, and herd health management strategies (99, 100).

### 4.3 Transmission dynamics in endemic systems

As discussed in Section 4.1, ASF and PRRS epidemiology in Asia diverges in the drivers of viral persistence, particularly in regions transitioning toward endemicity (Table 3). ASFV transmission is shaped by viral environmental resilience, stability in fomites that serve as pathogen carriers, thereby providing efficient indirect pathways (33). On the other hand, PRRSV transmission is characterized by high contagiousness, robust within-herd circulation, and greater reliance on direct and airborne pathways (101). ASFV airborne spread may occur over limited distances of 2–10 meters, but still requires further validation (102). Conversely, PRRSV possibly spreads via aerosols over longer distances, up to 3 kilometers under favorable atmospheric conditions (103). Regarding waterborne transmission, improper burial of carcasses or river disposal allows ASFV to leach into shallow aquifers, where it can remain infectious for over 42 days at 4 °C or 14 days at 21 °C (104). In contrast, the fragile PRRSV degrades rapidly during carcass decomposition and survives in groundwater for less than 11 days, presenting a much lower, highly localized transmission risk (105).

At the herd level, both viruses can spread directly (snout-to-snout contact) or indirectly (vehicle-borne), vertically from sows to offspring (more common with PRRSV than with ASFV), and through behaviors such as biting, fighting (higher risk with ASFV, which is blood-borne, compared to PRRSV, which is primarily transmitted via saliva) (64). These routes allow stable infection cycles, typically in farrow-to-finish systems where age groups overlap and sustain PRRSV and low-virulence/chronic ASFV (except for high-virulence isolates, which rapidly deplete host herds). Unique to PRRS, incomplete herd immunity, viral diversity, and the introduction of susceptible or non-acclimatized animals facilitate silent persistence or emergence of new variants (106).

At the regional level, both viruses have been documented to seed novel outbreaks via long-haul incidents, such as imported contaminated boar semen or infected trade pigs, or breeding stock (31, 107–109). In addition, the role of wild species is discussed in Section 3.4; the wild boar serves as a key reservoir for ASFV and classical swine fever (CSF) virus, compared to PRRSV in the domestic pig population (110–112). Pests or insects can carry pathogens from ASFV/PRRSV-contaminated materials between farms (52). Unique to ASFV, soft ticks are biological hosts (virus is able to propagate inside) in the sylvatic cycle, but they might not play an important role as vectors in Asia, probably because of the relative abundance of hard ticks compared to soft ticks or the paucity of studies focusing on vector-borne ASF outbreaks (113). There is no evidence that non-swine species can serve as biological vectors for PRRSV, but houseflies and mosquitoes may facilitate its mechanical transmission (114, 115).

In brief, although ASFV and PRRSV share certain transmission routes within the Asian production system, ASFV dissemination is predominantly driven by human-mediated activities rather than by direct animal-to-animal contact alone, while PRRSV dissemination relies on efficient host-to-host transmission and viral evolution, enabling a self-sustaining endemic infection system. Effective control, therefore, hinges on disrupting within-herd transmission rather than solely preventing external introductions, as with ASFV.

### 4.4 Diagnostic approaches

Diagnostic approaches for ASFV and PRRSV reflect their contrasting epidemiologies but pose practical challenges where both co-circulate. ASFV diagnosis prioritizes rapid, individual confirmation of acute infection via polymerase chain reaction (PCR)-based assays targeting the conserved *B646L* (p72 gene), demonstrating high sensitivity (>98%) in whole blood, tissues, and swabs within 2 weeks post-infection. Antibody detection in sera via enzyme-linked immunosorbent assay (ELISA) is ineffective for acute triage because rapid mortality precedes seroconversion (after day 10); therefore, it is the primary tool for herd-level surveillance to identify surviving carriers of low-virulence or gene-deleted strains that exhibit intermittent shedding (116). However, as endemicity transforms, lower viral loads and intermittent shedding may reduce PCR sensitivity, complicating detection in apparently healthy animals. Consequently, ASFV diagnostic strategies are shifting from acute single-animal clearance toward population-level herd surveillance frameworks modeled after PRRSV monitoring programs.

Conversely, the PRRSV diagnostic is structured for continuous, population-level herd monitoring to maintain herd stability rather than for single-animal clearance. It requires a combined molecular and serological approach due to its endemic nature and complex infection dynamics (117). Active viremia is routinely monitored using reverse-transcription (RT)-qPCR in serum, oral fluids, or processing fluids, while serology assesses herd exposure, immunity, and vaccination status via ELISA or, less likely, serum neutralization (SN), with ELISA more commonly used as a low-cost, high-throughput method compared to SN (118). However, a positive serology result does not distinguish between vaccination and field infection or cross-protection among strains (119).

The field confirmation of natural ASFV and PRRSV co-infections, with up to 1.12% (35/3,116 samples) dual-positivity in pig farms, slaughterhouses, and harmless treatment plants, creates diagnostic interference and strains veterinary infrastructure in resource-limited zones (120, 121). To resolve these field ambiguities, genomic surveillance has become indispensable. Molecular epidemiology relies on sequencing the ORF5 gene, which represents less than 5% of the PRRSV genome. Consequently, ORF5 analysis is blind to recombination events occurring in non-structural polyproteins, frequently misclassifying highly virulent recombinant field strains or MLV-revertants as benign parental lineages (66). Next-generation WGS provides the ultimate resolution to these limitations. For PRRSV, WGS is the only definitive method to map precise intra- and inter-lineage recombination breakpoints (83). For ASFV, WGS differentiates highly homologous G.II variants, traces transboundary pathways, and distinguishes natural field strains from engineered gene-deleted live vaccines (122, 123).

### 4.5 National and regional strategy responses

National and regional responses to ASF and PRRS in East Asia and SEA are converging toward consistency. This reflects a shift from effort-based eradication models toward adaptive coexistence through risk-based management and immunization that align with each country's contextual realities rather than a single control paradigm.

In the fight against ASF, during the epidemic, all countries sought to rapidly control and prevent viral spread and, eventually, eradicate the disease. Response plans primarily focuses on regional zoning, restrictions on live-animal and pork-product movement, a swill-feeding ban, enhanced diagnosis and surveillance, re-/depopulation, strengthened biosecurity measures, and production system consolidation (17, 124–126). However, the outcomes have not been as expected during recent sporadic outbreaks, even though the swine industry has recovered to pre-ASF baselines in some countries (Figure 1). As ASF suddenly disrupted the pork market and has recently become endemic, national control strategies have shifted away from strict eradication toward more adaptive approaches that balance disease control with production continuity and food security. For instance, a massive culling protocol has been replaced by a precision culling (colloquially termed the “tooth-extraction” protocol), defined as the early molecular detection, isolation, and localized removal of only infected pigs, applied in several countries (e.g., China, Vietnam, the Philippines) to curtail financial losses and preserve non-infected stock (127, 128).

Across the region, individual strategies are highly dependent on domestic production structure, veterinary institutional capacity, financial compensation frameworks, law enforcement and penalties, and policy priorities (Table 3).

China demonstrates a state-driven, industrialization-based model, in which it continues to explore compartmentalization, implementation of timely, effective, and accurate emergency responses, sectoral restructuring, and improve workforce training to support large-scale containment and recovery (129). The country has pioneered the implementation of the “tooth-extraction” protocol (127). Structurally, the industry has expanded into “multi-story,” defined as highly consolidated, multi-level, climate-controlled swine facilities. These facilities utilize modern physical infrastructure, including multi-stage air filtration networks and automated, zero-contact feeding workflows to minimize animal-pathogen contact (130).

Vietnam represents a combined approach, using biological tools with partial biosecurity improvements and establishing disease-free compartmentalization to manage its highly diverse farm systems (125). It was the first nation to officially authorize commercial live-attenuated vaccines (LAVs) for ASF, beginning large-scale rollouts to address widespread endemic circulation. However, this strategy faces distinct challenges regarding vaccine efficacy and safety (90). Because small-scale farmers face high financial risks from vaccine-associated adverse events, the cost of vaccination, and variable confidence, vaccine coverage remains low, as evidenced by only 4 million doses used compared to over 30 million head of the national pig population (131). This uneven coverage, combined with the unmonitored field use of live vaccines, has introduced severe evolutionary pressure. Indeed, ASF vaccine-like variants have been detected and circulated in the non-vaccinated population, associated with a chronic-like infection (122, 123). In addition, the available commercial LAVs for G.II have not protected against emerging rI/II strains (132).

Thailand delayed reporting its initial ASF introduction stemming from socioeconomic sensitivities to disease disclosure in a leading pork-producing and exporting nation (133, 134). Another contributing factor may be the structural focus on veterinary infrastructure within the Thai private swine sectors, supported by the Thai Swine Veterinary Association (TSVA) and complemented by a centralized system of livestock production governance. This top-heavy system likely overlooked the informal household sectors, where it is economically unfeasible with lower capital for physical biosecurity barriers, and reliance on informal trading networks resulted in hotspots for the first ultimate outbreaks (126). The national plan cautiously adopts a biosecurity-centered containment strategy at the farm level, with stronger regulatory enforcement and limited reliance on vaccination, thereby restricting compartmentalized transmission. Government initiatives have performed advanced practical training programs and issued farm certification (e.g., mandatory Good Agricultural Practices-GAP), targeting medium- to large-scale farmers (135, 136). These efforts have allowed Thailand to maintain a controlled endemic state characterized by sporadic, localized outbreaks rather than widespread epidemic waves (126).

In contrast, the Philippines illustrates a fragmented endemic scenario characterized by archipelagic geography, with standard strategies and integrated vaccination. The ASF conventional control measure “1–7–10” defines infected (1 km), buffer (7 km), and surveillance (10 km) zones (17). To overcome the challenges faced by smallholders, who frequently hide outbreaks due to fear of uncompensated culling, the government launched an intensive, a three-phase repopulation program—comprising depopulation, cleaning, and disinfection (30 days), downtime (20 days), and sentinel animals (40 days)—that was validated and implemented (137). Since 2024, government-led vaccination programs have been rolling out commercial vaccines, expanding from pilots to widespread use by 2025–2026, with approximately 350 thousand doses administered (138, 139). Thus far, distinct scenarios across the three main islands of the Philippines demonstrate the ongoing risks of inter-island spread and re-emergence, as shown in recent pork production data (Figure 1) (140). By contrast, Taiwan successfully claimed ASF-free status months after the first detection, thanks to swift, decisive responses. These include zoning and culling all pigs within 3-km restricted areas, completely stopping animal and product movement, banning swill feeding, and inspecting 100% passenger luggage at the ports (141). Taiwan may have been facilitated by its island geography, which inherently restricts transboundary animal movement, together with effective coordination among stakeholders and veterinary authorities and sustained public education efforts.

PRRS control has centered on long-term endemic management through early detection and monitoring, viral load reduction, routine vaccination, inter-farm biosecurity, and herd-level stabilization (142). Similar to current ASF-endemic scenarios, the effectiveness of PRRS management strategies depends on industry structure, veterinary facilities, and control capacity, and tends to be adaptive rather than elimination-focused. China applies coordinated, large-scale herd stabilization within industrial systems through the “load-close-homogenize” (LCH) practices and/or repopulates breeding herds through “testing, removal, and rollover” strategies (143, 144). Vietnam relies on increasing the vaccination coverage on medium- and large-scale farms and partial biosecurity in smallholder-dominated settings, following recommendations from a simulation model analysis (145). Thailand achieves more stable control through integrated production and stricter biosecurity measures, such as all-in/all-out management, gilt acclimatization, and strategic vaccine timing to maintain stable herds or PRRSV-free herds, compared with other countries in the region, even though PRRSV continues to circulate in mixed systems (146). The Philippines faces disjointed, inconsistent control, particularly in households, due to decentralized systems and limited resources for surveillance, biosecurity, and coordinated interventions (147). Hence, most East and SEA countries have been attempting these actions to address the ongoing ASF endemicity.

It should be noted that, after WOAH approved Taiwan's self-declaration of ASF-free status by April 6, 2026, following the single outbreak reported in October 2025 (148), Taiwan is the only country in Asia free from three major swine diseases: ASF, FMD, and CSF (17). Nonetheless, the detection of PRRSV-2 lineage 1 in Taiwan represents the first documented evidence of the introduction of a foreign lineage into the country through breeder import, followed by its subsequent dissemination across the entire island. It was speculated to be a silent introduction, with unremarkable clinical signs observed, leading to the omission of appropriate testing (149). The outcomes of the two diseases differ substantially depending on the level of awareness and the extent of regulatory authorization. In particular, when authorities prioritize ASF control and implement transparent reporting and response strategies, effective disease control can be achieved. Globally, these selected actions align with WOAH's principles of risk-based management, compartmentalization, and progressive control for primary eradication. Asia shows that flexible, context-specific strategies are vital not only for ASF and PRRS but also for other TADs in diverse settings. However, several knowledge gaps and challenges remain unsolved, requiring further research and optimization.

### 4.6 Knowledge gaps and strategic priorities

ASFV and PRRSV present two distinct paradigms regarding epidemiological knowledge gaps and management strategies. ASFV is considered a knowledge-limited disease, characterized by significant uncertainties in transmission, environmental persistence, and host-pathogen interactions. Compounded by the absence of effective diagnostic control tools, particularly with the emerging low-virulence/chronic strains, this results in low predictability in epidemiological dynamics and a heavy reliance on biosecurity-based approaches (150). In contrast, PRRSV benefits from available vaccines and management strategies, but control remains inconsistent. This can be explained in part by high viral variability, leading to vaccines' incomplete/failed protection against heterologous infection in pigs, complicated transmission dynamics, and uneven implementation across production systems (97). Thus, ASFV gaps stem from fundamental unknowns, whereas PRRSV gaps reflect challenges in optimizing and applying existing knowledge, though the PRRS immune response remains a work in progress as viral evolution progresses (151). These contrasting profiles define distinct research and control priorities, as summarized in Table 4.

**Table 4:** Knowledge gaps comparison between ASF and PRRS.

Aspect	Priority and domain	ASF	PRRS	Comparative insights
Diagnostics and surveillance	Critical urgency Applied research	Limited early detection for low-virulence strains; weak surveillance in smallholders; field diagnostics need improvement (60, 120, 162, 168)	Difficulty distinguishing vaccine vs. field strains (unavailable DIVA tools); limited real-time tracking tools (119, 165)	ASF: sensitivity gap for chronic, hidden carriers PRRS: interpretation barrier due to vaccine-field strain cross-reactions
Vaccines	High urgency Applied research	No fully reliable vaccine; safety concerns with LAVs; lack of DIVA strategies (122, 131, 154)	Existing vaccines have poor cross-protection; risk of reversion, viral shifts; limited efficacy against diverse strains (151, 153, 156)	ASF: safety, efficacy vaccine absence PRRS: vaccine performance problem
Control and biosecurity	High urgency Field application	Unclear feasibility of eradication vs. control; low compliance in smallholder systems (125, 140, 163)	Herd stabilization strategies exist but not scalable; inconsistent regional success (6, 142, 145)	ASF: strategy feasibility gap PRRS: strategy scalability gap
Viral biology and evolution	High urgency Fundamental research	Limited functional genomics; unclear virulence determinants; poorly understood within-host evolution and adaptation (91, 168)	Recombination dynamics not fully resolved; weak genotype-phenotype predictability; vaccine-driven evolution mechanism unclear (77, 144, 169)	ASF: lack of basic functional genomics regarding viral plasticity understanding PRRS: lack predictive evolutionary models for hyper-mutagenic shifts
Pathogenesis and immunology	Moderate urgency Fundamental research	No clear correlates of protection; immune response poorly defined; chronic infection mechanisms unclear (160, 170)	Immune evasion known, but correlates of protection unclear, incomplete vaccine immunity; co-infection effects unresolved (e.g., ASFV + PRRSV) (10, 80, 95, 151)	Both pathogens lack defined protective correlates; immunological understanding more limited in ASF
Cross-cutting gaps	Moderate urgency Field application	Lack of standardized epidemiological metrics; limited integration of production systems; weak regional datasets (3, 5, 141)	Same as ASF; plus insufficient integration of vaccination and endemic management strategies (4, 98, 100)	Shared failure to integrate localized veterinary data into unified, system-level epidemiological network in region
Transmission dynamics	Low-to-Moderate urgency Field application	Unclear role of indirect pathways, wild boar and vector-borne (soft ticks); environmental persistence vs. actual transmission unclear (51, 150)	Aerosol recognized but poorly quantified; persistence via subclinical carriers (wild boar) not fully understood; herd-level maintenance unclear (65, 103, 111)	ASF: mechanistic and biological uncertainty; multi-host complexity PRRS: quantitative data gap for lateral aerosol spread; within-host persistence complexity

Across affected East and SEA systems, the interrelated knowledge gaps and challenges are ranked below by operational domain (ongoing research vs. practical field application), urgency, and potential epidemiological impact:

*Domain A: high-urgency fundamental and applied research*

(1) Diagnostic efficiency and surveillance gaps for deletion mutant: standard surveillance tools lack sensitivity for subclinical and low-virulence ASFV infections caused by emerging gene-deleted strains (e.g., Δ*MGF* or Δ*EP402R* mutants) due to low viral loads and overlapping clinical presentations in PRRS-endemic backgrounds. This sometimes creates an early-detection sensitivity gap for ASF and a diagnostic interpretation gap for PRRSV, thereby permitting silent viral persistence (120, 152).
(2) Vaccine safety and companion differentiating infection and vaccination animals (DIVA) tools: the unmonitored field deployment of unauthorized or unvalidated LAVs and MLVs poses severe biosecurity risks in endemic areas lacking robust monitoring. This amplifies the emergence of vaccine-derived variants, wild-type recombinants, and reversion-to-virulence mutants, making the development of reliable DIVA tools an absolute research priority (122, 153, 154).
(3) Accelerating viral genetic diversity: the rapid field expansion of naturally occurring low-virulent, recombinant, and vaccine-like strains leaves within-host evolution, adaptation, and transmission dynamics unclear (60, 90, 155). This escalating genetic diversity continuously challenges the cross-protection of commercial vaccines, particularly against mutating PRRSV lineages or hybrid rI/II ASFV strains (132, 156).
(4) Pathogenesis of clinical co-infections: natural co-infection between these two diseases adds complexity to pathogenesis and clinical outcomes. This dual-infection challenge amplifies herd mortality, causes severe economic devastation, and reduces the effectiveness of conventional single-pathogen herd-stabilization protocols (120, 121).

*Domain B: high-impact scalable field application*

(5) Socio-economic and structural constraints: fragmented smallholder production networks face severe structural constraints, including low biosecurity compliance and weak traceability (3). For ASF, the primary practical bottleneck is the economic unfeasibility of enforcing mandatory herd-fallowing and depopulation periods among smallholders (140). For PRRSV, the limitation lies in a failure to translate academic knowledge into context-appropriate, scalable multi-site herd-stabilization protocols (e.g., LCH) across non-integrated farms (142, 143).
(6) Value-chain disinfection pathways: indirect transmission routes and human-mediated mechanical transfer across formal and informal livestock networks remain poorly managed. Enforcing targeted, verifiable sanitation and vehicle-disinfection protocols at key transit hubs and wet markets represents a highly effective practical intervention in complex cross-border and island systems (157).
(7) Micro-epidemiology and vector ecology: gaps persist in predicting localized airborne PRRSV transmission, understanding within-farm persistence mechanisms, and defining the true transmission risk posed bywildlife reservoirs, such as wild boars, and biological vectors, such as *Ornithodoros* soft ticks (97, 150). Mitigating these multi-host pathways represents a lower immediate practical priority than securing anthropogenic marketing networks (157).

All in all, future research should prioritize identifying the epidemiological dynamics of subclinical ASF infections and reliable detection tools, such as DIVA methods; validating vaccine safety and monitoring frameworks; linking viral diversity to epidemiological outcomes; and characterizing transmission pathways within complex production and trade networks (Table 4). Equally critical are studies addressing implementation barriers, including socio-economic drivers and biosecurity incentives, to ensure that existing and emerging control tools are effectively and uniformly applied across the regions' diverse production systems.

## 5 Conclusion

This narrative review characterized and contrasted the distinct molecular and transmission mechanisms of ASFV and PRRSV. It demonstrated that while these pathogens present divergent biological profiles, they exploit identical structural and socio-economic vulnerabilities within high-density networks. The comparative insight of this study revealed that ASFV primarily operates as an anthropogenic, human-driven disease; its relatively slow within-herd transmission is offset by the extreme environmental stability of inanimate objects, which facilitates prolonged mechanical persistence. Conversely, PRRSV functions as a self-sustaining, host-persistent, endemic engine driven by high within-herd contagiousness and long-distance, aerosol-mediated lateral transmission. Crucially, the evolutionary patterns of both viruses have been converging as East and SEA transition into an endemic landscape. The field emergence of high-lethality rI/II ASFV strains and vaccine-like microvariants closely mirrored the extensive intra- and inter-lineage genomic recombination long documented for PRRSV, resulting in complex clinical co-infections that heavily strain existing regional veterinary diagnostic and regulatory infrastructure. Together, both diseases underscore that effective preparedness for TAD prevention and control requires not only scientific advances but also alignment across national contexts.

In evaluating transboundary viral dissemination, this study discussed how regional variations in pig housing, geopolitical connectivity, culinary cultures, and live-animal marketing structures accelerate transmission across China, Vietnam, Thailand, and the Philippines. Fragmented supply chains and porous borders facilitate the spread of both pathogens, rendering traditional, reactive national control strategies, such as mass stamping-out policies, economically unfeasible and unsustainable. Appraising the successes and failures of existing interventions demonstrated that while formal compartment systems and zoning offer theoretical protection, their execution is frequently undermined by the realities of informal value chains. Furthermore, the persistence of PRRSV despite widespread commercial vaccination highlighted clear implementation gaps in contrast to the highly variable efficacy and safety challenges surrounding newly authorized field control measures for ASFV. To shift regional frameworks toward resilient disease preparedness, future strategies must replace mass culling with validated selective culling in smallholder settings, while implementing strict herd management schemes like LCH and air-filtered containment within integrated industrial operations. On a macro-level, WGS is preferred for monitoring molecular epidemiological dynamics (e.g., mutants, recombinants), and cross-border surveillance must be harmonized through regional bodies like the Global Framework for the Progressive Control of Transboundary Animal Diseases (GF-TADs), led by WOAH and FAO, to regulate live-animal and pork-derived product movements.

While addressing its primary objectives, several inherent limitations constrained the definitive scope of this review. First, the evolution of national control strategies and marketing structures was challenged by heterogeneous domestic data systems, with highly variable data recording frameworks and livestock classifications, which preclude direct quantitative standardization of farm-scale market impacts across the region. Second, the geographical scope of the granular analysis was intentionally restricted to a strategic cross-section of four major producing nations. Consequently, specific localized risk pathways and transboundary dynamics unique to other sub-regions may not be fully represented. Lastly, the literature selection strategy relied primarily on English-language, peer-reviewed publications indexed in major scientific databases, thereby omitting potentially valuable localized reports/conferences/meetings, field white papers, and primary native-language reports issued by domestic agricultural authorities within the studied nations.

## References

[1] VanderWaal K Deen J. Global trends in infectious diseases of swine. Proc Natl Acad Sci. (2018) 115:11495–500. doi: 10.1073/pnas.1806068115 30348781 PMC6233110

[2] Mai TN Nguyen TT Dang-Xuan S Nguyen-Viet H Unger F Lee HS. Transboundary viral diseases of pigs, poultry and ruminants in southeast asia: a systematic review. Vet Q. (2024) 44:13–25. doi: 10.1080/01652176.2024.2397796 39210836 PMC11370669

[3] Wang L Li D. Current status, challenges and prospects for pig production in asia. Anim Biosci. (2024) 37:742. doi: 10.5713/ab.23.0303 38419542 PMC11016695

[4] Kedkovid R Sirisereewan C Thanawongnuwech R. Major swine viral diseases: an Asian perspective after the African swine fever introduction. Porcine Health Manag. (2020) 6:20. doi: 10.1186/s40813-020-00159-x 32637149 PMC7336096

[5] Woonwong Y Do Tien D Thanawongnuwech R. The future of the pig industry after the introduction of african swine fever into asia. Anim Front. (2020) 10:30–7. doi: 10.1093/af/vfaa037 33150009 PMC7596796

[6] Assavacheep P Thanawongnuwech R. Porcine respiratory disease complex: dynamics of polymicrobial infections and management strategies after the introduction of the African swine fever. Front Vet Sci. (2022) 9:1048861. doi: 10.3389/fvets.2022.1048861 36504860 PMC9732666

[7] WOAH. African Swine Fever (Infection with African Swine Fever Virus). Manual of Diagnostic Tests and Vaccines for Terrestrial Animals. Paris: World Organization for Animal Health (2025).

[8] Dixon LK Stahl K Jori F Vial L Pfeiffer DU. African swine fever epidemiology and control. Annu Rev Anim Biosci. (2020) 8:221–46. doi: 10.1146/annurev-animal-021419-083741 31743062

[9] WOAH. African Swine Fever (Asf)—Situation Report. Paris: WOAH (2026).

[10] Lunney JK Fang Y Ladinig A Chen N Li Y Rowland B . Porcine reproductive and respiratory syndrome virus (Prrsv): pathogenesis and interaction with the immune system. Annu Rev Anim Biosci. (2016) 4:129–54. doi: 10.1146/annurev-animal-022114-111025 26646630

[11] Shin J Kang Y Kim Y Yeom S Kweon C Lee W . Sero-epidemiological studies on porcine reproductive and respiratory syndrome in Korea. I Detection of Indirect Fluorescent Antibodies RDA J Agri Sci. (1993) 35:572–6.

[12] Production-Pork [Internet]. (2026). Available online at: https://www.fas.usda.gov/data/production/0113000 (Accessed March 8, 2026).

[13] Meat Comsumption [Internet]. (2026). Available online at: https://www.oecd.org/en/data/indicators/meat-consumption.html (Accessed March 8, 2026).

[14] Huynh T Aarnink A Drucker A Verstegen M. Pig production in Cambodia, Laos, Philippines, and Vietnam: a review. Asian J Agric Dev (2006) 4: 69–90. doi: 10.37801/ajad2006.3.1-2.5

[15] Thanapongtharm W Linard C Chinson P Kasemsuwan S Visser M Gaughan AE . Spatial analysis and characteristics of pig farming in Thailand. BMC Vet Res. (2016) 12:218. doi: 10.1186/s12917-016-0849-7 27716322 PMC5053203

[16] Zhao Q Dupas M-C Axelsson C Artois J Robinson TP Gilbert M. Distribution and intensification of pig production in China 2007–2017. Environ Res Lett. (2022) 17:124001. doi: 10.1088/1748-9326/aca16b

[17] Hsu C-H Chang C-Y Otake S Molitor TW Perez A. Strategies for transboundary swine disease management in asian islands: foot and mouth disease, classical swine fever, and African swine fever in Taiwan, Japan, and the Philippines. Vet Sci. (2024) 11:130. doi: 10.3390/vetsci11030130 38535864 PMC10973987

[18] USDA. (2025) *Livestock and Products Annual. Country Report*. September 19, 2025. Report No: RP2025-0038.

[19] NSO. Agriculture, Forestry and Fishing (2023) .Available online at: https://www.nso.gov.vn/en/statistical-data/ (Accessed March 12, 2026)

[20] DLD. Livestock Animal in Thailand. Bangkok: Department of Livestock Development (2022).

[21] Alarcón LV Allepuz A Mateu E. Biosecurity in Pig Farms: A review. Porcine Health Manag. (2021) 7:5. doi: 10.1186/s40813-020-00181-z 33397483 PMC7780598

[22] Mutua F Dione M. The context of application of biosecurity for control of African swine fever in smallholder pig systems: current gaps and recommendations. Front Vet Sci. (2021) 8:689811. doi: 10.3389/fvets.2021.689811 34409087 PMC8364973

[23] Lamberga K Olševskis E SerŽants M Bērzinš A Viltrop A Depner K. African swine fever in two large commercial pig farms in latvia—estimation of the high risk period and virus spread within the farm. Vet Sci. (2020) 7:105. doi: 10.3390/vetsci7030105 32784575 PMC7559702

[24] Dietz K. The estimation of the basic reproduction number for infectious diseases. Stat Methods Med Res. (1993) 2:23–41. doi: 10.1177/096228029300200103 8261248

[25] Pittman Ratterree DC Chitlapilly Dass S Ndeffo-Mbah ML. The reproduction number of swine viral respiratory diseases: a systematic review. Vet Sci (2024) 11:300. doi: 10.3390/vetsci11070300 39057984 PMC11281358

[26] SRAT. Thailand's Pig Production on the Rise—but Prices Fall and Small-Scale Farmers Suffer (2025). Available online at: https://www.swinethailand.com/ (Accessed March 12, 2026).

[27] Gonsalves JF Carandang A Verallo III JR Barbon WJ. Asian Mega-Deltas (Amd): Derisking Delta-Oriented Value Chains in Cambodia, Vietnam and Myanmar: Scoping Study on Key Production Systems and Value Chains. Silang, Cavite (Philippines): International Institute of Rural Reconstruction (IIRR) (2022).

[28] McOrist S Khampee K Guo A. Modern pig farming in the People's Republic of China: growth and veterinary challenges. Rev Sci Tech. (2011) 30:961. doi: 10.20506/rst.30.3.2091 22435207

[29] Liu Z Zhu Z Wu Z Ning K. African swine fever, consumer confidence and pork consumption behaviors in China: a case study in Shanghai. China Agric Econ Rev. (2025) 17:825–41. doi: 10.1108/CAER-07-2024-0215

[30] Hu J-H Pei X Sun G-Q Jin Z. Risk analysis of the transmission route for the African swine fever virus in Mainland China. Front Phys. (2021) 9:785885. doi: 10.3389/fphy.2021.785885

[31] Gao X Liu T Liu Y Xiao J Wang H. Transmission of African swine fever in China through legal trade of live pigs. Transbound Emerg Dis. (2021) 68:355–60. doi: 10.1111/tbed.13681 32530109

[32] Bartolome MJ Aguirre LAM Poliquit CM Besas I Angeles JG Rabajante J . Environmental DNA (Edna) Contamination patterns of african swine fever virus (Asfv) in swine transport vehicles in the Philippines. Vet Med (Praha). (2025) 70:156. doi: 10.17221/84/2024-VETMED 40735303 PMC12303034

[33] Mazur-Panasiuk N Żmudzki J Wozniakowski G. African swine fever virus–persistence in different environmental conditions and the possibility of its indirect transmission. J Vet Res. (2019) 63:303. doi: 10.2478/jvetres-2019-0058 31572808 PMC6749736

[34] Ito S Bosch J Martínez-Avilés M Sánchez-Vizcaíno JM. The evolution of african swine fever in China: a global threat? Front Vet Sci. (2022) 9:828498. doi: 10.3389/fvets.2022.828498 35425825 PMC9001964

[35] Yen DA-w Cappellini B Wang CL Nguyen B. Food consumption when traveling abroad: young Chinese Sojourners' food consumption in the Uk. Appetite (2018) 121:198-206. doi: 10.1016/j.appet.2017.11.097 29154887

[36] Miller LP Miknis RA Flory GA. Carcass Management Guidelines: Effective Disposal of Animal Carcasses and Contaminated Materials on Small to Medium-Sized Farms. Rome: FAO (2020).

[37] Taylor KC. Environmental impacts of the foot and mouth disease outbreak in Great Britain in 2001: the use of risk analysis to manage the risks in the countryside. Rev Sci Tech (2002) 21:797–813. doi: 10.20506/rst.21.3.1375 12523716

[38] Dee SA Bauermann FV Niederwerder MC Singrey A Clement T de Lima M . Survival of Viral pathogens in animal feed ingredients under transboundary shipping models. PLoS ONE. (2018) 13:e0194509. doi: 10.1371/journal.pone.0194509 29558524 PMC5860775

[39] Shurson GC Urriola PE Schroeder DC. Biosecurity and mitigation strategies to control swine viruses in feed ingredients and complete feeds. Animals. (2023) 13:2375. doi: 10.3390/ani13142375 37508151 PMC10376163

[40] Boklund A Dhollander S Chesnoiu Vasile T Abrahantes JC Bøtner A Gogin A . Risk factors for African swine fever incursion in romanian domestic farms during 2019. Sci Rep. (2020) 10:10215. doi: 10.1038/s41598-020-66381-3 32576841 PMC7311386

[41] Li J Zhang J Gao L Nie B Zhu H. Evaluating the impact of swill-feeding on the control of African swine fever in China with a dynamic model. Chaos Soliton Fract. (2024) 186:115262. doi: 10.1016/j.chaos.2024.115262

[42] Dame-Korevaar A Boumans IJ Antonis AF van Klink E de Olde EM. Microbial health hazards of recycling food waste as animal feed. Future Foods. (2021) 4:100062. doi: 10.1016/j.fufo.2021.100062

[43] Maes D Van Soom A Appeltant R Arsenakis I Nauwynck H. Porcine semen as a vector for transmission of viral pathogens. Theriogenology. (2016) 85:27–38. doi: 10.1016/j.theriogenology.2015.09.046 26506911

[44] Bierk M Dee SA Rossow K Otake S Collins J Molitor T. Transmission of porcine reproductive and respiratory syndrome virus from persistently infected sows to contact controls. Can J Vet Res. (2001) 65:261. 11768134 PMC1189689

[45] Ito S Kawaguchi N Bosch J Aguilar-Vega C Sánchez-Vizcaíno JM. What can we learn from the five-year African swine fever epidemic in Asia? Front Vet Sci. (2023) 10:1273417. doi: 10.3389/fvets.2023.1273417 37841468 PMC10569053

[46] Xiaofang D Cansong L Xiaofeng L Yunchang R. Spatiotemporal process and operational mechanism of illegal cross-border cattle trade between China and Myanmar. Trop Geogr (2024) 44: 1184–1195. doi: 10.13284/j.cnki.rddl.20230771

[47] Aguilar-Vega C Sánchez-Vizcaíno JM Bosch J. Identifying sites where wild boars can consume anthropogenic food waste with implications for African swine fever. PLoS ONE. (2024) 19:e0308502. doi: 10.1371/journal.pone.0308502 39116050 PMC11309469

[48] Liao H Lyon CJ Ying B Hu T. Climate change, its impact on emerging infectious diseases and new technologies to combat the challenge. Emerg Microbes Infect. (2024) 13:2356143. doi: 10.1080/22221751.2024.2356143 38767202 PMC11138229

[49] Denstedt E Porco A Hwang J Nga NTT Ngoc PTB Chea S . Detection of African swine fever virus in free-ranging wild boar in Southeast Asia. Transbound Emerg Dis. (2021) 68:2669–75. doi: 10.1111/tbed.13964 33351995 PMC8518571

[50] Tu Z Sun H Wang T Liu Y Xu Y Peng P . Node role of wild boars in virus circulation among wildlife and domestic animals. Nat Commun. (2025) 16:8938. doi: 10.1038/s41467-025-64019-4 41062486 PMC12508043

[51] Kawaguchi N Aguilar-Vega C Sasaki M Orba Y Sawa H Sánchez-Vizcaíno JM . Risk mapping of African swine fever in domestic pigs and wild boars to enhance management and surveillance in Asia. Transbound Emerg Dis. (2025) 2025:8850856. doi: 10.1155/tbed/8850856 41323145 PMC12662683

[52] Makovska I Chantziaras I Dhaka P Courtens L Kox L Dewulf J. Flies and beetles-mediated transmission of pathogens in domestic pigs: a systematic review. J Insects Food Feed. (2025) 11:1339–56. doi: 10.1163/23524588-00001415

[53] Gaudreault NN Madden DW Wilson WC Trujillo JD Richt JA. African swine fever virus: an emerging DNA arbovirus. Front Vet Sci. (2020) 7:215. doi: 10.3389/fvets.2020.00215 32478103 PMC7237725

[54] Lazov CM Olesen AS Belsham GJ Bøtner A. Assessing virus survival in African swine fever virus-contaminated materials—implications for indirect virus transmission. Viruses. (2025) 17:63. doi: 10.3390/v17010063 39861852 PMC11769059

[55] Bastos AD Penrith M-L Cruciere C Edrich J Hutchings G Roger F . Genotyping field strains of African swine fever virus by partial P72 gene characterization. Arch Virol. (2003) 148:693–706. doi: 10.1007/s00705-002-0946-8 12664294

[56] Fiori MS Sanna D Scarpa F Floris M Di Nardo A Ferretti L . A deeper insight into evolutionary patterns and phylogenetic history of Asfv epidemics in Sardinia (Italy) through extensive genomic sequencing. Viruses (2021) 13:1994. doi: 10.3390/v13101994 34696424 PMC8539718

[57] Zhang Y Wang Q Zhu Z Wang S Tu S Zhang Y . Tracing the origin of genotype Ii African swine fever virus in China by genomic epidemiology analysis. Transbound Emerg Dis. (2023) 2023:4820809. doi: 10.1155/2023/4820809 40303812 PMC12017148

[58] Mighell E Ward MP. African swine fever spread across Asia, 2018–2019. Transbound Emerg Dis. (2021) 68:2722–32. doi: 10.1111/tbed.14039 33599077

[59] Sun E Huang L Zhang X Zhang J Shen D Zhang Z . Genotype I African swine fever viruses emerged in domestic pigs in China and caused chronic infection. Emerg Microbes Infect. (2021) 10:2183–93. doi: 10.1080/22221751.2021.1999779 34709128 PMC8635679

[60] Sun E Zhang Z Wang Z He X Zhang X Wang L . Emergence and prevalence of naturally occurring lower virulent African swine fever viruses in domestic pigs in China in 2020. Sci China Life Sci. (2021) 64:752–65. doi: 10.1007/s11427-021-1904-4 33655434

[61] Zhao D Sun E Huang L Ding L Zhu Y Zhang J . Highly lethal genotype I and Ii recombinant African swine fever viruses detected in pigs. Nat Commun. (2023) 14:3096. doi: 10.1038/s41467-023-38868-w 37248233 PMC10226439

[62] Sun Y Xu Z Gao H Xu S Liu J Xing J . Detection of a novel African swine fever virus with three large-fragment deletions in Genome, China. Microbiol Spectr. (2022) 10:e0215522. doi: 10.1128/spectrum.02155-22 36000903 PMC9603391

[63] Nguyen TVH Kim Y-H Nguyen VD Tran TCG Vu ND Vu TTH . Rapid spread of recombinant African swine fever virus genotypes I and Ii, Vietnam, 2023–2024. Emerg Infect Dis. (2026) 32:649. doi: 10.3201/eid3204.251688 41987262 PMC13094834

[64] Pileri E Mateu E. Review on the transmission porcine reproductive and respiratory syndrome virus between pigs and farms and impact on vaccination. Vet Res. (2016) 47:108. doi: 10.1186/s13567-016-0391-4 27793195 PMC5086057

[65] Allende R Laegreid WW Kutish GF Galeota JA Wills RW Osorio FA. Porcine reproductive and respiratory syndrome virus: description of persistence in individual pigs upon experimental infection. J Virol. (2000) 74:10834–7. doi: 10.1128/JVI.74.22.10834-10837.2000 11044133 PMC110963

[66] Shi M Lam TT Hon CC Murtaugh MP Davies PR Hui RK . Phylogeny-based evolutionary, demographical, and geographical dissection of North American type 2 porcine reproductive and respiratory syndrome viruses. J Virol. (2010) 84:8700–11. doi: 10.1128/JVI.02551-09 20554771 PMC2919017

[67] Li C Xu H Zhao J Gong B Sun Q Xiang L . Epidemiological Investigation and Genetic Evolutionary Analysis of Prrsv-1 on a Pig Farm in China. Front Microbiol. (2022) 13:1067173. doi: 10.3389/fmicb.2022.1067173 36532471 PMC9751794

[68] Xiang L Xu H Li C Tang YD An TQ Li Z . Long-term genome monitoring retraces the evolution of novel emerging porcine reproductive and respiratory syndrome viruses. Front Microbiol. (2022) 13:885015. doi: 10.3389/fmicb.2022.885015 35495717 PMC9044490

[69] Pamornchainavakul N Paploski IA Makau DN Kikuti M Rovira A Lycett S . Mapping the dynamics of contemporary prrsv-2 evolution and its emergence and spreading hotspots in the U.S. using phylogeography. Pathogens. (2023) 12:740. doi: 10.3390/pathogens12050740 37242410 PMC10222675

[70] Black EJ Powell CS Dempsey DM Hendrickson RC Mims LR Lefkowitz EJ. Virus taxonomy: the database of the international committee on taxonomy of viruses. Nucleic Acids Res. (2026) 54:D776–D89. doi: 10.1093/nar/gkaf1159 41296552 PMC12807731

[71] Yim-Im W Anderson TK Paploski IA VanderWaal K Gauger P Krueger K . Refining Prrsv-2 genetic classification based on global Orf5 sequences and investigation of their geographic distributions and temporal changes. Microbiol Spectr. (2023) 11:e02916–23. doi: 10.1128/spectrum.02916-23 37933982 PMC10848785

[72] VanderWaal K Pamornchainavakul N Kikuti M Linhares DC Trevisan G Zhang J . Phylogenetic-based methods for fine-scale classification of Prrsv-2 Orf5 sequences: a comparison of their robustness and reproducibility. Front Virol. (2024) 4:1433931. doi: 10.3389/fviro.2024.1433931

[73] VanderWaal K Pamornchainavakul N Kikuti M Zhang J Zeller M Trevisan G . Prrsv-2 Variant classification: a dynamic nomenclature for enhanced monitoring and surveillance. mSphere. (2025) 10:e0070924. doi: 10.1128/msphere.00709-24 39846734 PMC11852939

[74] Tian X Wei Z Khan M Zhou Z Zhang J Huang X . Refining lineage classification and updated Rflp patterns of Prrsv-2 revealed viral spatiotemporal distribution characteristics in China in 1991-2023. Transbound Emerg Dis. (2025) 2025:9977088. doi: 10.1155/tbed/9977088 40302738 PMC12017074

[75] Yim-Im W Anderson TK Böhmer J Baliellas J Stadejek T Gauger PC . Refining genetic classification of global porcine reproductive and respiratory syndrome virus type 1 (Prrsv-1) and investigating their geographic and temporal distributions. Vet Microbiol. (2025) 302:110413. doi: 10.1016/j.vetmic.2025.110413 39904077

[76] Stadejek T Oleksiewicz MB Scherbakov AV Timina AM Krabbe JS Chabros K . Definition of subtypes in the European genotype of porcine reproductive and respiratory syndrome virus: nucleocapsid characteristics and geographical distribution in Europe. Arch Virol. (2008) 153:1479–88. doi: 10.1007/s00705-008-0146-2 18592131

[77] Weng C Huang X Chen Z He M Zhang B Li H . Genetic evolution, epidemic trends, and recombination dynamics of Prrsv-1 in China. Front Vet Sci. (2025) 12:1632917. doi: 10.3389/fvets.2025.1632917 40838145 PMC12363365

[78] An T-Q Tian Z-J Leng C-L Peng J-M Tong G-Z. Highly pathogenic porcine reproductive and respiratory syndrome virus, Asia. Emerg Infect Dis. (2011) 17:1782. doi: 10.3201/eid1709.110411 21888830 PMC3322091

[79] Shi M Holmes EC Brar MS Leung FC-C. Recombination is associated with an outbreak of novel highly pathogenic porcine reproductive and respiratory syndrome viruses in China. J Virol. (2013) 87:10904–7. doi: 10.1128/JVI.01270-13 23885071 PMC3807407

[80] Liu J Liu C Xu Y Yang Y Li J Dai A . Molecular characteristics and pathogenicity of a novel recombinant porcine reproductive and respiratory syndrome virus strain from Nadc30-, Nadc34-, and Jxa1-like strains that emerged in China. Microbiol Spectr. (2022) 10:e02667–22. doi: 10.1128/spectrum.02667-22 36354339 PMC9769985

[81] Chen N Cao Z Yu X Deng X Zhao T Wang L . Emergence of novel European genotype porcine reproductive and respiratory syndrome virus in Mainland China. J Gen Virol (2011) 92(Pt 4):880–92. doi: 10.1099/vir.0.027995-0 21216986

[82] Wang X Yang X Zhou R Zhou L Ge X Guo X . Genomic characterization and pathogenicity of a strain of type 1 porcine reproductive and respiratory syndrome virus. Virus Res. (2016) 225:40–9. doi: 10.1016/j.virusres.2016.09.006 27619842

[83] Chen N Liu Q Qiao M Deng X Chen X Sun M. Whole genome characterization of a novel porcine reproductive and respiratory syndrome virus 1 isolate: genetic evidence for recombination between Amervac vaccine and circulating strains in Mainland China. Infect Genet Evol. (2017) 54:308–13. doi: 10.1016/j.meegid.2017.07.024 28746838

[84] Salguero FJ. Comparative pathology and pathogenesis of African swine fever infection in swine. Front Vet Sci. (2020) 7:282. doi: 10.3389/fvets.2020.00282 32509811 PMC7248413

[85] Piao S Jin X Hu S Lee J-Y. The impact of African swine fever on the efficiency of China's pig farming industry. Sustainability. (2024) 16:7819. doi: 10.3390/su16177819

[86] Nguyen-Thi T Pham-Thi-Ngoc L Nguyen-Ngoc Q Dang-Xuan S Lee HS Nguyen-Viet H . An assessment of the economic impacts of the 2019 African swine fever outbreaks in Vietnam. Front Vet Sci. (2021) 8:686038. doi: 10.3389/fvets.2021.686038 34760953 PMC8573105

[87] Cheng J Ward MP. Risk factors for the spread of African swine fever in China: a systematic review of Chinese-language literature. Transbound Emerg Dis. (2022) 69:e1289–e98. doi: 10.1111/tbed.14573 35490407 PMC9790558

[88] Shao Q Li R Han Y Han D Qiu J. Temporal and spatial evolution of the African swine fever epidemic in Vietnam. Int J Environ Res Public Health. (2022) 19:8001. doi: 10.3390/ijerph19138001 35805660 PMC9265385

[89] Nguyen TT Venkateswaran D Suntisukwattana R Jongkaewwattana A Thaweerattanasinp T Saenboonrueng J . First Detection of a Recombinant African Swine Fever Virus Gi/Ii in Domestic Pigs from a Border Area near Thailand. Chulalongkorn University Veterinary Conference. Bangkok: Thai J Vet Med (2026).

[90] Nguyen TC Tantitaveewattana P Pamornchainavakul N Do DT Kedkovid R Thanawongnuwech R. Subtle genetic shifts of African swine fever virus among Vietnamese domestic swine following live-attenuated vaccine commercialization. Transbound Emerg Dis. (2026) 2026:8776906. doi: 10.1155/tbed/8776906 41789191 PMC12956841

[91] O'Dwyer J Van HV Phuong NT Mileto P Mercado O. Conceição Fd, et al. Emergence of microvariants of African swine fever virus genotype Ii in the Asia–Pacific. Transbound Emerg Dis. (2025) 2025:9990044. doi: 10.1155/tbed/9990044 40585857 PMC12204745

[92] Montecillo AD Baybay ZK Ferrer JBC Cariaso W Pantua A Jose JP . Genetic profiles of ten African swine fever virus strains from outbreaks in select provinces of Luzon, Visayas, and Mindanao, Philippines, between 2021 and 2023. Viruses. (2025) 17:588. doi: 10.3390/v17040588 40285030 PMC12031577

[93] Salman M Venkateswaran D Prakash A Nguyen QA Suntisukwattana R Atthaapa W . The comparative full-length genome characterization of African swine fever virus detected in Thailand. Animals. (2024) 14:2602. doi: 10.3390/ani14172602 39272387 PMC11394130

[94] Shi K Liu H Yin Y Si H Long F Feng S. Molecular characterization of African swine fever virus from 2019–2020 outbreaks in Guangxi Province, Southern China. Front Vet Sci. (2022) 9:912224. doi: 10.3389/fvets.2022.912224 35782548 PMC9240437

[95] Chand RJ Trible BR Rowland RR. Pathogenesis of porcine reproductive and respiratory syndrome virus. Curr Opin Virol. (2012) 2:256–63. doi: 10.1016/j.coviro.2012.02.002 22709514

[96] Perez AM Davies PR Goodell CK Holtkamp DJ Mondaca-Fernández E Poljak Z . Lessons learned and knowledge gaps about the epidemiology and control of porcine reproductive and respiratory syndrome virus in North America. J Am Vet Med Assoc. (2015) 246:1304–17. doi: 10.2460/javma.246.12.1304 26043128

[97] Montaner-Tarbes S Del Portillo HA Montoya M Fraile L. Key gaps in the knowledge of the porcine respiratory reproductive syndrome virus (Prrsv). Front Vet Sci. (2019) 6:38. doi: 10.3389/fvets.2019.00038 30842948 PMC6391865

[98] Zhang Z Li Z Li H Yang S Ren F Bian T . The economic impact of porcine reproductive and respiratory syndrome outbreak in four Chinese farms: based on cost and revenue analysis. Front Vet Sci. (2022) 9:1024720. doi: 10.3389/fvets.2022.1024720 36311672 PMC9597626

[99] Boeters M Garcia-Morante B Van Schaik G Segalés J Rushton J Steeneveld W. The economic impact of endemic respiratory disease in pigs and related interventions-a systematic review. Porcine Health Manag. (2023) 9:45. doi: 10.1186/s40813-023-00342-w 37848972 PMC10583309

[100] Nathues H Alarcon P Rushton J Jolie R Fiebig K Jimenez M . Modeling the economic efficiency of using different strategies to control porcine reproductive & respiratory syndrome at herd level. Prev Vet Med. (2018) 152:89–102. doi: 10.1016/j.prevetmed.2018.02.005 29559110

[101] Otake S Yoshida M Dee S. A review of swine breeding herd biosecurity in the United States to prevent virus entry using porcine reproductive and respiratory syndrome virus as a model pathogen. Animals (2024) 14:2694. doi: 10.3390/ani14182694 39335283 PMC11440104

[102] Li X Hu Z Fan M Tian X Wu W Gao W . Evidence of aerosol transmission of African swine fever virus between two piggeries under field conditions: a case study. Front Vet Sci. (2023) 10:1201503. doi: 10.3389/fvets.2023.1201503 37323846 PMC10267313

[103] Arruda AG Tousignant S Sanhueza J Vilalta C Poljak Z Torremorell M . Aerosol detection and transmission of porcine reproductive and respiratory syndrome virus (Prrsv): what is the evidence, and what are the knowledge gaps? Viruses. (2019) 11:712. doi: 10.3390/v11080712 31382628 PMC6723176

[104] Loundras EA Netherton CL Flannery J Bowes MJ Dixon L Batten C. The effect of temperature on the stability of African swine fever virus Ba71v isolate in environmental water samples. Pathogens (2023) 12:1022. doi: 10.3390/pathogens12081022 37623982 PMC10459264

[105] Lugo Mesa V Quinonez Munoz A Sobhy NM Corzo CA Goyal SM. Survival of porcine reproductive and respiratory syndrome virus (Prrsv) in the environment. Vet Sci (2024) 11:22. doi: 10.3390/vetsci11010022 38250928 PMC10820812

[106] Lambert M-È Denicourt M Poljak Z D'Allaire S. Gilt replacement strategies used in two swine production areas in Quebec in regard to porcine reproductive and respiratory syndrome virus. J Swine Health Prod. (2012) 20:223–30. doi: 10.54846/jshap/719

[107] Nathues C Perler L Bruhn S Suter D Eichhorn L Hofmann M . An outbreak of porcine reproductive and respiratory syndrome virus in Switzerland following import of boar semen. Transbound Emerg Dis. (2016) 63:e251–e61. doi: 10.1111/tbed.12262 25209832

[108] Zhou L Wang Z Ding Y Ge X Guo X Yang H. Nadc30-like strain of porcine reproductive and respiratory syndrome virus, China. Emerg Infect Dis. (2015) 21:2256. doi: 10.3201/eid2112.150360 26584305 PMC4672414

[109] Friedrichs V Reicks D Hasenfuß T Gerstenkorn E Zimmerman JJ Nelson EA . Artificial insemination as an alternative transmission route for African swine fever virus. Pathogens. (2022) 11:1539. doi: 10.3390/pathogens11121539 36558873 PMC9785317

[110] Cadenas-Fernández E Ito S Aguilar-Vega C Sánchez-Vizcaíno JM Bosch J. The role of the wild boar spreading African swine fever virus in Asia: another underestimated problem. Front Vet Sci. (2022) 9:844209. doi: 10.3389/fvets.2022.844209 35573420 PMC9093143

[111] Choi E-J Lee C-H Hyun B-H Kim J-J Lim S-I Song J-Y . Survey of porcine reproductive and respiratory syndrome among wild boar populations in Korea. J Vet Sci. (2012) 13:377. doi: 10.4142/jvs.2012.13.4.377 23271179 PMC3539123

[112] Ito S Jurado C Bosch J Ito M Sánchez-Vizcaíno JM Isoda N . Role of wild boar in the spread of classical swine fever in Japan. Pathogens (2019) 8:206. doi: 10.3390/pathogens8040206 31653072 PMC6963481

[113] Luskin MS Meijaard E Surya S. Sheherazade, Walzer C, Linkie M. African swine fever threatens Southeast Asia's 11 endemic wild pig species. Conserv Lett. (2021) 14:e12784. doi: 10.1111/conl.12784

[114] Otake S Dee SA Rossow KD Moon RD Pijoan C. Mechanical transmission of porcine reproductive and respiratory syndrome virus by mosquitoes, aedes vexans (meigen). Can J Vet Res. (2002) 66:191–5. 12146891 PMC227003

[115] Pitkin A Deen J Otake S Moon R Dee S. Further Assessment of Houseflies (Musca Domestica) as Vectors for the Mechanical Transport and Transmission of Porcine Reproductive and Respiratory Syndrome Virus under Field Conditions. Can J Vet Res. (2009) 73:91−6. 19436589 PMC2666325

[116] Hu Z Tian X Lai R Wang X Li X. Current detection methods of African swine fever virus. Front Vet Sci. (2023) 10:1289676. doi: 10.3389/fvets.2023.1289676 38144466 PMC10739333

[117] Henao-Diaz A Ji J Giménez-Lirola L Baum DH Zimmerman J. Understanding and interpreting Prrsv diagnostics in the context of “disease transition stages.” *Res Vet Sci*. (2020) 131:173–6. doi: 10.1016/j.rvsc.2020.04.023 32388019

[118] Ge M Li RC Gong W Tu C. Determination of antibody induction by highly pathogenic porcine reproductive and respiratory syndrome virus (Hp-Prrsv) vaccine: a comparison of two Elisa kits. J Vet Med Sci. (2019) 81:1173–6. doi: 10.1292/jvms.18-0482 31189757 PMC6715930

[119] Pan J Zeng M Zhao M Huang L. Research progress on the detection methods of porcine reproductive and respiratory syndrome virus. Front Microbiol. (2023) 14:1097905. doi: 10.3389/fmicb.2023.1097905 36970703 PMC10033578

[120] Rajkhowa S Sonowal J Pegu SR Sanger GS Deb R Das PJ . Natural co-infection of pigs with African swine fever virus and porcine reproductive and respiratory syndrome virus in India. Braz J Microbiol. (2024) 55:1017−22. doi: 10.1007/s42770-023-01203-y 38041718 PMC10920511

[121] Feng Z Shi K Yin Y Shi Y Feng S Long F . A quadruplex Rt-Qpcr for the detection of African swine fever virus, classical swine fever virus, porcine reproductive and respiratory syndrome virus, and porcine pseudorabies virus. Animals (2024) 14:3551. doi: 10.3390/ani14233551 39682516 PMC11640363

[122] Nguyen TC Bui NTT Nguyen LT Ngo TNT Van Nguyen C Nguyen LM . An African swine fever vaccine-like variant with multiple gene deletions caused reproductive failure in a vietnamese breeding herd. Sci Rep. (2025) 15:14919. doi: 10.1038/s41598-025-95641-3 40295549 PMC12037777

[123] Nguyen TT Venkateswaran D Prakash A Nguyen QA Suntisukwattana R Jongkaewwattana A . Vaccine-like African swine fever virus strain in domestic pigs, Thailand, 2024. Emerg Infect Dis. (2026) 32:299. doi: 10.3201/eid3202.251245 41715192 PMC12928227

[124] Li J Zhang J Gao L Li S Zhu H. Dynamical modeling and analysis of the impact of zonal prevention and control under normalized management on African swine fever transmission in China. Infect Dis Model. (2026) 11:1325–1343. doi: 10.1016/j.idm.2026.03.011 42205148 PMC13202274

[125] DAH. African Swine Fever Situation and Swine Disease Free Zones in Viet Nam (2024). Available online at: https://rr-asia.woah.org/app/uploads/2024/12/7.3-ASF-and-ADF-zones-in-Viet-Nam.pdf (Accessed March 24, 2026).

[126] DLD. Asf in Thailand: Biosecurity Approach (2023). Available online at: https://rr-asia.woah.org/app/uploads/2023/08/2-10_sge-asf-country-update-on-biosecurity-approach-thailand.pdf (Accessed March 29, 2026).

[127] Du X Liu Y Duan L Tsai S-Y Yaros JP Wu F. Elimination of Asfv via precise culling in a large-scale breeding herd in China: a field experience. Animals. (2025) 15:2521. doi: 10.3390/ani15172521 40941316 PMC12427241

[128] Nga BTT Padungtod P Depner K Chuong VD Duy DT Anh ND . Implications of partial culling on African swine fever control effectiveness in Vietnam. Front Vet Sci. (2022) 9:957918. doi: 10.3389/fvets.2022.957918 36118335 PMC9479321

[129] Wang G. Asf in China: Current Status, Control Measures and Experience (2023). Available online at: https://www.woah.org/app/uploads/2023/04/12-4-gongmin-wang-slides.pdf (Accessed March 30, 2026).

[130] Ma J Chen H Gao X Xiao J Wang H. African swine fever emerging in China: distribution characteristics and high-risk areas. Prev Vet Med. (2020) 175:104861. doi: 10.1016/j.prevetmed.2019.104861 31810030

[131] Auer A Cattoli G Padungtod P Lamien CE Oh Y Jayme S . Challenges in the application of African swine fever vaccines in Asia. Animals (2024) 14:2473. doi: 10.3390/ani14172473 39272258 PMC11393951

[132] Diep NV Duc NV Ngoc NT Dang VX Tiep TN Nguyen VD . Genotype Ii live-attenuated Asfv vaccine strains unable to completely protect pigs against the emerging recombinant Asfv genotype I/Ii strain in Vietnam. Vaccines. (2024) 12:1114. doi: 10.3390/vaccines12101114 39460281 PMC11511409

[133] Songkasupa T Dokphut A Boonpornprasert P. Detection of African swine fever virus in confiscated pork products brought into Thailand during 2018–2019. In: 19th Chulalongkorn University Veterinary Conference (CUVC 2020). Bangkok (2020) 50:257−9.

[134] Thanapongtharm W Wiratsudakul A Gilbert M Chamsai T Pabutta C Wiriyarat W . Spatial prediction of wild boar distribution in Thailand applications for African swine fever prevention and control. Sci Rep. (2025) 15:9987. doi: 10.1038/s41598-025-94922-1 40121282 PMC11929826

[135] DLD. Swine Industry and Swine Disease Control in Thailand (2020). Available online at: https://rr-asia.woah.org/app/uploads/2020/06/2-3_thailand.pdf (Accessed March 29, 2026).

[136] TSVA. Clinical Practice Guideline for Uplevel Biosecurity on African Swine Fever. Bangkok: Thai Swine Veterinary Association (TSVA) (2022).

[137] Hsu C-H Montenegro M Miclat-Sonaco R Torremorell M Perez AM. Validation of the effectiveness of pig farm repopulation protocol following African swine fever outbreaks in the Philippines. Front Vet Sci. (2024) 11:1468906. doi: 10.3389/fvets.2024.1468906 39717795 PMC11663853

[138] Fernandez-Colorado CP Kim WH Flores RA Min W. African swine fever in the Philippines: a review on surveillance, prevention, and control strategies. Animals. (2024) 14:1816. doi: 10.3390/ani14121816 38929435 PMC11200829

[139] MAE. Vietnam Exports 340,000 Doses of African Swine Fever Vaccine to the Philippines. Hanoi: Ministry of Agriculture and Environment Vienam (2025). Available online at: https://en.mae.gov.vn/vietnam-exports-340000-doses-of-african-swine-fever-vaccine-to-the-philippines-8997.htm

[140] Hsu C-H Schambow RA Humphreys J Montenegro M Arzt J Perez AM. Perspective: challenges and research opportunities to enhance African swine fever control in the Philippines. Front Vet Sci. (2025) 12:1675095. doi: 10.3389/fvets.2025.1675095 41099063 PMC12518057

[141] Woo PCY Chuang K-C Chen M-H Chen C-M. Emergence of African swine fever in Taiwan: epidemiology and control strategies. EMI: Anim Environ. (2026) 2:2643811. doi: 10.1080/29986990.2026.2643811

[142] Rowland RR Morrison RB. Challenges and opportunities for the control and elimination of porcine reproductive and respiratory syndrome virus. Transbound Emerg Dis. (2012) 59 (Suppl 1):55–9. doi: 10.1111/j.1865-1682.2011.01306.x 25471243

[143] Hu Y Zhao K Wu G Hong H Xia T Liu Z . Combining load-close-homogenize with testing, removal, and rollover strategies to repopulate Prrsv elimination breeding herds using Prrsv-positive weaned gilts. Vet Sci (2025) 12:1012. doi: 10.3390/vetsci12101012 41150152 PMC12567787

[144] Guo Z Chen XX Li R Qiao S Zhang G. The prevalent status and genetic diversity of porcine reproductive and respiratory syndrome virus in China: a molecular epidemiological perspective. Virol J. (2018) 15:2. doi: 10.1186/s12985-017-0910-6 29301547 PMC5753475

[145] Lee HS Thakur KK Bui VN Bui AN Dang MV Wieland B. Simulation of control scenarios of porcine reproductive and respiratory syndrome in Nghe an Province in Vietnam. Transbound Emerg Dis. (2019) 66:2279–87. doi: 10.1111/tbed.13278 31233273 PMC6899877

[146] TSVA. Clinical Practice Guideline (Cpg) for Prrs in Thailand. Bangkok: Thai Swine Veterinary Association (TSVA) (2015).

[147] Abao LNB. Economic analysis of the impact of infectious pig diseases and its control in Philippine (dissertation). Obihiro University of Agriculture and Veterinary Medicine, Obihiro (2014).

[148] OCAC. Taiwan Regains Self-Declared Asf-Free Status from Woah (2026) [updated April 7, 2026]. Available online at:https://www.taipeitimes.com/News/taiwan/archives/2026/04/07/2003855168 (Accessed April 10, 2026).

[149] Kuo KL Lin WH Chiou MT Zhang J Lin CN. Co-circulation of lineage 1 and lineage 3 porcine reproductive and respiratory syndrome virus type 2 (Prrsv-2) in Taiwan during 2018–2024. Vet Microbiol. (2025) 306:110567. doi: 10.1016/j.vetmic.2025.110567 40414106

[150] Authority EFS Nielsen SS Alvarez J Bicout DJ Calistri P Depner K . Research priorities to fill knowledge gaps in the control of African swine fever: possible transmission of African swine fever virus by vectors. EFSA J. (2021) 19:e06676. doi: 10.2903/j.efsa.2021.6676 34188718 PMC8215588

[151] Li J Miller LC Sang Y. Current status of vaccines for porcine reproductive and respiratory syndrome: interferon response, immunological overview, and future prospects. Vaccines (2024) 12:606. doi: 10.3390/vaccines12060606 38932335 PMC11209547

[152] Kwon T Bae G-S Jeon E Kang P Son HC An YJ . Global challenges and advancements in the management of pivotal porcine/swine viral diseases. In Vivo. (2025) 39:1810–32. doi: 10.21873/invivo.13982 40578970 PMC12223619

[153] Pamornchainavakul N Paploski IA Makau DN Baker JP Huang J Ferreira CP . Experimental evidence of vaccine-driven evolution of porcine reproductive and respiratory syndrome virus type 2. Virus Evol (2025) 11:veaf056. doi: 10.1093/ve/veaf056 40831531 PMC12360701

[154] van den Born E Olasz F Mészáros I Göltl E Oláh B Joshi J . African swine fever virus vaccine strain Asfv-G-ΔI177l reverts to virulence and negatively affects reproductive performance. NPJ Vaccines. (2025) 10:46. doi: 10.1038/s41541-025-01099-9 40050309 PMC11885574

[155] Trevisan G Magstadt D Woods A Sparks J Zeller M Li G . A recombinant porcine reproductive and respiratory syndrome virus type 2 field strain derived from two Prrsv-2-modified live virus vaccines. Front Vet Sci. (2023) 10:1149293. doi: 10.3389/fvets.2023.1149293 37056231 PMC10086154

[156] Raev SA Cai L Muro N Madera R Wang L Shi J. Cross-protection in Prrsv: mechanisms, limitations, and implications for vaccine design. Pathogens. (2026) 15:345. doi: 10.3390/pathogens15040345 42075672 PMC13119244

[157] Beltran-Alcrudo D Falco JR Raizman E Dietze K. Transboundary spread of pig diseases: the role of international trade and travel. BMC Vet Res. (2019) 15:64. doi: 10.1186/s12917-019-1800-5 30795759 PMC6387505

[158] Sun Q Xu H An T Cai X Tian Z Zhang H. Recent progress in studies of porcine reproductive and respiratory syndrome virus 1 in China. Viruses (2023) 15:1528. doi: 10.3390/v15071528 37515213 PMC10384046

[159] Guinat C Gubbins S Vergne T Gonzales JL Dixon L Pfeiffer DU. Experimental pig-to-pig transmission dynamics for African swine fever virus, Georgia 2007/1 strain. Epidemiol Infect. (2016) 144:25–34. doi: 10.1017/S0950268815000862 25989921 PMC4697298

[160] Ruedas-Torres I Thi To Nga B Salguero FJ. Pathogenicity and virulence of African swine fever virus. Virulence. (2024) 15:2375550. doi: 10.1080/21505594.2024.2375550 38973077 PMC11232652

[161] You S Liu T Zhang M Zhao X Dong Y Wu B . African swine fever outbreaks in China led to gross domestic product and economic losses. Nat Food. (2021) 2:802–8. doi: 10.1038/s43016-021-00362-1 37117973

[162] Chuong VD Schambow RA Diep NT Minh PQ Long NV To Nga BT . Epidemiology and control of African swine fever in Vietnam: a scoping review. Pathogens. (2025) 14:329. doi: 10.3390/pathogens14040329 40333166 PMC12030036

[163] WOAH. Asean African Swine Fever (Asf) Prevention and Control Strategy (2023-−*2028)* (2023). Available online at: https://rr-asia.woah.org/app/uploads/2024/12/ASEAN_ASF_Strategy_Final.pdf (Accessed April 2, 2026).

[164] Ngo TTN Oh T Do DT. The prospects and challenges of live attenuated vaccines against African swine fever virus in Vietnam. Vaccines. (2026) 14:284. doi: 10.3390/vaccines14030284 41893820 PMC13030726

[165] Thanawongnuwech R Suradhat S. Taming Prrsv: revisiting the control strategies and vaccine design. Virus Res. (2010) 154:133–40. doi: 10.1016/j.virusres.2010.09.003 20851723

[166] Li C Fan A Liu Z Wang G Zhou L Zhang H . Prevalence, time of infection, and diversity of porcine reproductive and respiratory syndrome virus in China. Viruses. (2024) 16:774. doi: 10.3390/v16050774 38793655 PMC11125865

[167] Jantafong T Saenglub W Chaisilp N Paungpin W Tibkwang T Mutthi P . Investigation of the distribution and origin of porcine reproductive and respiratory syndrome virus 1 in the swine production chain: a retrospective study of three farms in Thailand. Vet World. (2024) 17:1722. doi: 10.14202/vetworld.2024.1722-1732 39328441 PMC11422652

[168] Chen S Wang T Luo R Lu Z Lan J Sun Y . Genetic variations of African swine fever virus: major challenges and prospects. Viruses (2024) 16:913. doi: 10.3390/v16060913 38932205 PMC11209373

[169] Trevisan G Sparks J Zeller M Tong H Li G Zhang J . Emergence of a Prrsv strain recombined from two modified-live virus vaccines and its elimination from a breeding herd. Transbound Emerg Dis. (2025) 2025:5770608. doi: 10.1155/tbed/5770608 40727310 PMC12301085

[170] Li M Zheng H. Insights and progress on epidemic characteristics, pathogenesis, and preventive measures of African swine fever virus: a review. Virulence (2025) 16(1):2457949. doi: 10.1080/21505594.2025.2457949 39937724 PMC11901552

